# Supplementation with α-ketoglutarate improved the efficacy of anti-PD1 melanoma treatment through epigenetic modulation of PD-L1

**DOI:** 10.1038/s41419-023-05692-5

**Published:** 2023-02-28

**Authors:** Nian Liu, Jianglin Zhang, Mingjie Yan, Lihui Chen, Jie Wu, Qian Tao, Bei Yan, Xiang Chen, Cong Peng

**Affiliations:** 1 Department of Dermatology, Xiangya Hospital, Central South University, Changsha, China; 2 Furong Laboratory, Xiangya Hospital, Central South University, Changsha, China; 3 Hunan Key Laboratory of Skin Cancer and Psoriasis, Human Engineering Research Center of Skin Health and Disease, Xiangya Hospital, Central South University, Changsha, China; 4 National Clinical Research Center for Geriatric Disorders, Xiangya Hospital, Central South University, Changsha, China; 5 Department of Dermatology, 2nd Clinical Medical College of Jinan University, Changsha, China; 6 Department of Clinical Pharmacology, Xiangya Hospital, Central South University, Changsha, China

**Keywords:** Drug development, Melanoma

## Abstract

Patients with advanced melanoma have shown an improved outlook after anti-PD1 therapy, but the low response rate restricts clinical benefit; therefore, enhancing anti-PD1 therapeutic efficacy remains a major challenge. Here, our findings showed a significantly increased abundance of α-KG in healthy controls, anti-PD1-sensitive melanoma-bearing mice, and anti-PD1-sensitive melanoma patients; moreover, supplementation with α-KG enhanced the efficacy of anti-PD1 immunotherapy and increased PD-L1 expression in melanoma tumors via STAT1/3. We also found that supplementation with α-KG significantly increased the activity of the methylcytosine dioxygenases TET2/3, which led to an increased 5-hydroxymethylcytosine (5-hmC) level in the PD-L1 promoter. As a consequence, STAT1/3 binding to the PD-L1 promoter was stabilized to upregulate PD-L1 expression. Importantly, single-cell sequencing of preclinical samples and analysis of clinical data revealed that TET2/3-STAT1/3-CD274 signaling was associated with sensitivity to anti-PD1 treatment in melanoma. Taken together, our results provide novel insight into α-KG’s function in anti-PD1 treatment of melanoma and suggest supplementation with α-KG as a novel promising strategy to improve the efficacy of anti-PD1 therapy.

## Introduction

Metabolic reprogramming is a hallmark of cancer, resulting in altered metabolites that facilitate tumorigenesis by altering the tumor microenvironment or gene expression [1]. α-ketoglutarate (α-KG, also known as 2-oxoglutarate) is a key intermediate metabolite in the tricarboxylic acid cycle and plays an important role in epigenetic function by acting as a cofactor for ten-eleven translocation (TET) enzymes [2, 3], which induce DNA 5-methylcytosine catalysis into 5-hydroxymethylcytosine in regulatory regions [4]. Three members of the TET family (TET1-3) have been identified, all of which are α-KG-dependent dioxygenases that contribute to DNA demethylation; however, the members that function in different tumors vary [5]. α-KG metabolic dysregulation can directly contribute to the progression of lymphoid and myeloid neoplasms by inhibiting α-KG-dependent TET dioxygenases [6]. Moreover, 5-hmC is downregulated in solid tumors, including breast cancer, colon cancer, and lung cancer [7, 8]; however, the role of α-KG-TET signaling in melanoma has not been clearly elucidated.

Cutaneous melanoma is an aggressive malignant tumor derived from melanocytes [9] that does not respond to traditional treatments, including chemotherapy and radiotherapy [10]. Given the high frequency of BRAF mutations, targeted BRAF inhibitors (BRAFis), such as vemurafenib and dabrafenib, were developed for advanced melanoma therapy; however, patients treated with a BRAFi eventually experience drug resistance [11]. Recently, the application of anti-PD1 treatment has been well-documented to improve the survival of patients with metastatic melanoma [12]. Unfortunately, the record response rate is less than 30%, and these patients are prone to acquired drug resistance [13, 14]; therefore, the identification of key pathways that can be targeted to improve the efficacy of PD1 blockade remains necessary.

Metabolites of the TCA cycle have been documented to significantly impact antitumor immunity and immunotherapy. Excess pyruvate in macrophages increases PD-L1 expression by enhancing oxidative phosphorylation and TCA cycle activity [15]. Glutamate increases PD-L1 expression by enhancing the TCA cycle, and disruption of glutamine metabolism enhances the therapeutic efficacy of PD1 blockade in activated B-cell-diffuse large B-cell lymphoma [16].

In this research, we demonstrated that a PD1-responsive group had dramatically increased levels of α-KG; furthermore, exogenous supplementation with α-ketoglutarate enhanced the efficacy of PD1 blockade, providing a novel promising therapeutic strategy for melanoma.

## Results

### The abundance of α-ketoglutarate was related to the efficacy of anti-PD1 mAb treatment

To elucidate the metabolic alterations in melanoma, NMR was performed to detect differences in the levels of plasma metabolites between 20 normal individuals and 20 melanoma patients, and that of α-KG was found to be significantly altered. As shown in Supplementary Fig. 1A, B, the level of α-KG was dramatically reduced in melanoma patient plasma, and the AUC value of α-KG was 0.945 (Supplementary Fig. 1A, B). To explore the role of α-KG in melanoma, we treated melanoma cells with membrane-permeable dm-α-KG at several concentrations. As shown in Supplementary Fig. 1C–F, the proliferation and clonogenic ability of melanoma cells were not altered after treatment with dm-α-KG over a wide range of concentrations in vitro, indicating that α-KG might not influence melanoma cell growth.

It was reported that Yumm1.7 cells are more sensitive to anti-PD1 treatment. Consistent with this result, we found that the Yumm1.7 tumor volume of mice was significantly reduced after treatment with an anti-PD1 antibody (Supplementary Fig. 2A); and that the percentage of tumor shrinkage after anti-PD1 treatment was significantly higher in Yumm1.7 tumor-bearing mice than in B16F10 tumor-bearing mice (Supplementary Fig. 2B). The percentage of CD45^+^ immune cells in Yumm1.7-derived tumors was significantly higher than that in B16F10-derived tumors, indicating that Yumm1.7-derived tumors had favorable immune cell infiltration (Supplementary Fig. 2C). Interestingly, significant upregulation of α-KG was found in the plasma of Yumm1.7 tumor-bearing mice, compared with that of B16F10 tumor-bearing mice (Supplementary Fig. 2D); Similarly, among melanoma patients treated with an anti-PD1 monoclonal antibody as an adjuvant therapy, the α-KG level was significantly higher in the patients who responded to anti-PD1 treatment than in the non-responders, and these results suggested that α-KG might have an important role in anti-PD1 immunotherapy (Supplementary Fig. 2E).

### Supplementation with α-ketoglutarate enhanced the efficacy of PD1 blockade

**Fig. 1:**
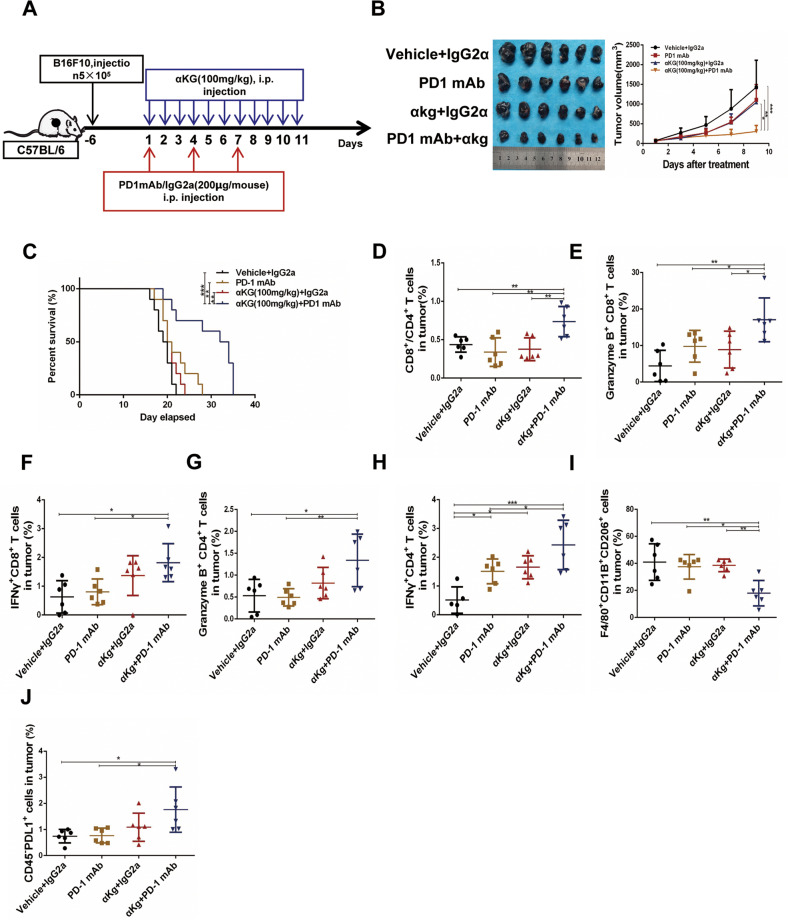
Supplementation with α-ketoglutarate treatment enhanced the efficacy of PD-1 blockade in B16F10 tumor-bearing mice. **A** Schematic diagram of α-ketoglutarate combined with PD1 mAb for melanoma treatment. **B** Tumors isolated from B16F10 tumor-bearing mice receiving the designated treatments (left panel). Tumor growth curves for B16F10 tumor-bearing mice receiving the designated treatments (right panel). **C** Survival curves of B16F10 tumor-bearing C57BL/6 mice. **D** The percentage of CD3^+^CD8^+^/CD3^+^CD4^+^ T cells in tumors determined by FACS. **E**–**I** The percentage of CD3^+^CD8^+^Granzyme B^+^ T cells **E**, CD3^+^CD8^+^IFNγ^+^ T cells **F**, CD3^+^CD4^+^Granzyme B^+^ T cells **G**, CD3^+^CD4^+^IFNγ^+^ T cells **H**, and F4/80^+^CD11B^+^MHC-II^+^ macrophages **I** in tumors determined by FACS. **J** The percentage of CD45^-^PDL1^+^ cells in tumors determined by FACS. Multiple experimental data (*n* = 6) are counted and presented according to the statistical methods, and an asterisk (*) indicates the degree of significant difference.

To determine whether α-KG contributes to the efficacy of PD1 blockade in melanoma, we treated B16F10 tumor-bearing mice with an anti-PD1 mAb, α-KG, α-KG plus the anti-PD1 mAb or corn oil plus an IgG isotype control (vehicle) (Fig. 1A). Treatment with α-KG or the anti-PD1 mAb alone did not significantly suppress melanoma growth. However, α-KG plus the anti-PD1 mAb significantly enhanced the therapeutic effect on B16F10 tumor-bearing mice (Fig. 1B). Similarly, α-KG strengthened the efficacy of anti-PD1 therapy in Yumm1.7 tumor-bearing mice that were sensitive to anti-PD1 treatment (Supplementary Fig. 3A). No obvious changes were observed in body weight, which suggested that the α-KG and anti-PD1 mAb treatments had limited toxicity in tumor-bearing mice (Supplementary Fig. 3B). More importantly, α-KG + anti-PD1 treatment significantly extended the overall survival time of the two mouse models (Fig. 1C, Supplementary Fig. 3C). To investigate the status of immune infiltration associated with the enhanced therapeutic efficacy of α-KG + anti-PD1, tumor-infiltrating leukocytes were analyzed at the termination of treatment. As shown in Fig. 1D, the ratio of CD8^+^/CD4^+^ T cells was significantly elevated in the α-KG + anti-PD1 group, indicating a strengthened immune reaction (Fig. 1D). In addition, we investigated the cytotoxicity of CD8^+^ and CD4^+^ T cells through the detection of granzyme B (GZMB) and interferon γ (IFNγ) expression in these cells. As shown in Fig. 1E–H, compared to tumors given control treatment, tumors treated with α-KG + anti-PD1 exhibited increased ratios of infiltrating GZMB^+^CD8^+^/CD4^+^ T cells and IFNγ^+^CD8^+^/CD4^+^ T cells, suggesting that α-KG supported the efficacy of the anti-PD1 mAb by enhancing the cytotoxicity of CD8^+^/CD4^+^ T cells. In addition, we discovered significant changes in M2 (F4/80^+^CD11b^+^CD206^+^) macrophage subsets, which represent a suppressive immune microenvironment [17] (Fig. 1E–H). As shown in Fig. 1I, we noticed a reduction in the level of immunosuppressive M2 macrophages in the α-KG + anti-PD1 group (Fig. 1I). Notably, the proportion of CD45^-^PDL1^+^ cells was elevated in the α-KG + anti-PD1 group, suggesting that α-KG enhanced the efficacy of the anti-PD1 mAb by promoting PD-L1 expression (Fig. 1J). However, no obvious changes were observed in the populations of MDSCs and Treg cells, indicating that the effect of α-KG + anti-PD1 on immunity was not related to these two immunosuppressive cell types (Supplementary Fig. 3D, E). In summary, our results showed that α-KG + anti-PD1 treatment increased the expression of PD-L1 in tumor cells and induced a tumor-killing immune microenvironment. These results indicate that α-KG supplementation may be a promising therapeutic strategy to enhance the efficacy of anti-PD1 mAb therapy in melanoma.

### α-Ketoglutarate improved anti-PD1 immunotherapy through IFNγ-STAT1/3-CD274 signaling

**Fig. 2:**
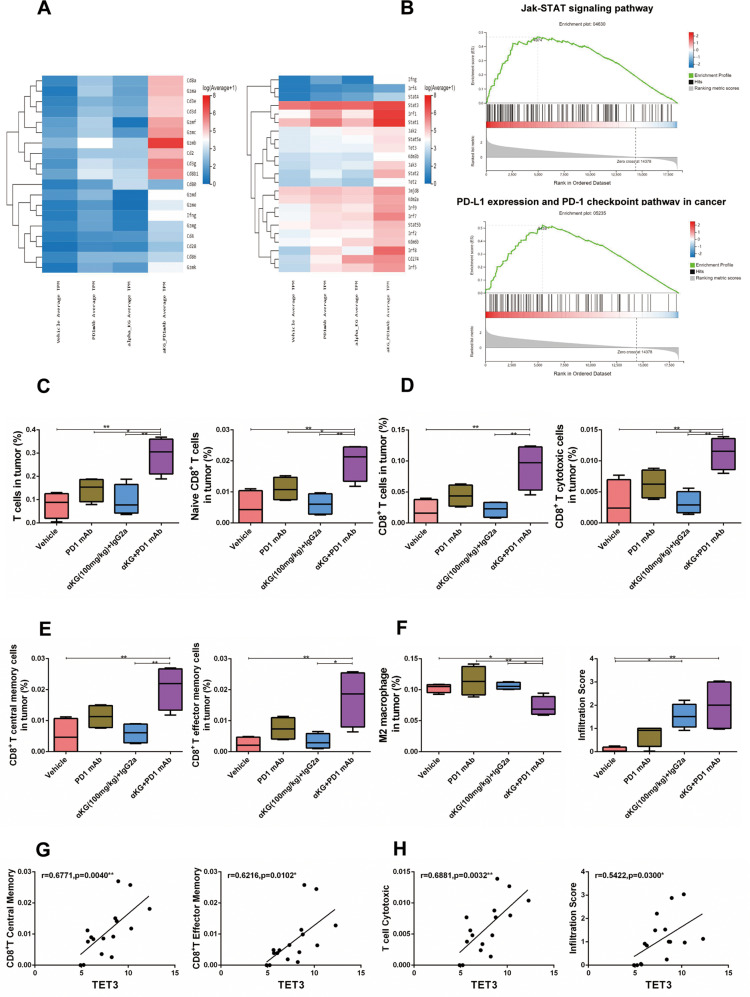
RNA-seq analyses of the effect of α-ketoglutarate and anti-PD-1 treatment on the gene expression profile. **A** Representative differential gene clustering map. **B** Enrichment map of GSEA signaling pathway enriched in α-KG + anti-PD1 group. C–F RNA-sequencing results were analyzed by the ImmuneCellAI-mouse method to characterize the immune microenvironment of melanoma tissue. The relative proportions of T cells (**C**, left panel), naive CD8^+^ T cells (**C**, right panel), CD8^+^ T cells (**D**, left panel), cytotoxic CD8^+^ T cells (**D**, right panel), central memory CD8^+^ T cells (**E**, left panel), effector memory CD8^+^ T cells (**E**, right panel) and M2 macrophages (**F**, left panel) and immune infiltration scores (**F**, right panel) in the tumor microenvironment. **G**, **H** Correlations of TET3 gene expression with the proportion of central memory CD8^+^ T cells (**G**, left panel), effector memory CD8^+^ T cells (**G**, right panel), cytotoxic CD8^+^ T cells (**H**, left panel) or immune infiltration scores (**H**, right panel). Multiple experimental data (*n* = 4) are counted and presented according to the statistical methods, and an asterisk (*) indicates the degree of significant difference.

Accumulated evidence suggests a key role for PD-L1 expression in anti-PD1 therapy [18]. To investigate the specific molecular mechanism by which α-KG enhances the efficacy of anti-PD1 therapy, RNA-seq was performed to profile the transcriptomic alterations in tumor tissues from B16F10 tumor-bearing mice after α-KG and anti-PD-1 mAb treatment, and 5633 significantly altered genes were identified (Supplementary Fig. 4A). Further analysis demonstrated that a number of the significantly altered genes were involved in multiple pathways, including T-cell-related pathways, cancer pathways, and JAK-STAT signaling pathways, according to their Kyoto Encyclopedia of Genes and Genomes pathway annotations (Supplementary Fig. 4B). We then performed cluster enrichment analysis of representative genes that were differentially expressed in these pathways (Fig. 2A) and observed that genes related to T-cell function, such as IFNG, GZMB, CD3, and CD8a, were significantly upregulated after combination therapy. In addition, genes related to the IFNγ-STAT-CD274 signaling pathway were found to be significantly altered (Fig. 2A), and gene set enrichment analysis (GSEA) confirmed the pathway enrichment of IFNγ signaling (JAK-STAT pathway) and PD1-PD-L1–related signaling pathways in the α-KG + anti-PD1 group (Fig. 2B). Further protein–protein interaction (PPI) analysis revealed that the major interacting factors were closely associated with IFNγ-STAT1/3-CD274 signaling (Supplementary Fig. 4C). We then performed transcription factor analysis and verified that the most significantly altered transcription factor was associated with the IFNγ-STAT signaling pathway (Supplementary Fig. 4D). Given these results, we hypothesized that supplementation with α-KG strengthens T-cell function by enhancing IFNγ-STAT1/3-CD274 signaling, which ultimately improves the therapeutic efficacy of anti-PD1 treatment.

To verify our speculation, we used the ImmuneCellAI-mouse method to measure the immune cell composition [19], and the results showed that the level of T cells, naive CD8^+^ T cells, CD8^+^ T cells, cytotoxic CD8^+^ T cells, central memory CD8^+^ T cells and effector memory CD8^+^ T cells were significantly increased in the α-KG + anti-PD1 group (Fig. 2C–E). Moreover, the level of immunosuppressive M2 macrophages was significantly decreased after α-KG + anti-PD1 treatment (Fig. 2F). Overall analysis of immune scores revealed that the immune infiltration score was highest in the combination group (Fig. 2F). These results indicated that the immune microenvironment was dramatically improved after α-KG plus anti-PD1 therapy, which was consistent with the previous results observed by flow cytometric analysis of the tumor.

Nevertheless, how α-KG affects IFNγ-STAT1/3-CD274 signaling remained unclear. Previous studies have demonstrated that α-KG acts as a cofactor of TET enzymes to increase the hydroxymethylation activity of these enzymes and remove methyl groups to increase gene expression [6]. α-KG can act as a cofactor for histone demethylases, which regulate gene expression by reducing the trimethylation of H3K9 and H3K27 [20]. According to the RNA-seq results, α-KG-dependent TET2/3 and histone demethylation genes were significantly altered in the α-KG + anti-PD1 group compared with the other groups (Fig. 2A, right panel). In contrast, H3K9me3 and H3K27me3, which are typical repressive marks that limit gene expression [21], were not affected by α-KG (Supplementary Fig. 5A, B), suggesting that α-KG influences IFNγ-STAT1/3-CD274 signaling through TET2/3. To test our hypothesis, we assessed the relationship among TET2/3, STAT-CD274 signaling, and the immune microenvironment. We observed that central memory CD8^+^ T cells, effector memory CD8^+^ T cells, T-cell toxicity and immune infiltration scores were positively correlated with the expression of TET3 (Fig. 2G, H). Similarly, TET2 expression was highly correlated with central memory CD8^+^ T cells, effector memory CD8^+^ T cells and T-cell toxicity (Supplementary Fig. 5C). Moreover, STAT1 and STAT3 were significantly correlated with effector memory CD8^+^ T cells, T-cell toxicity and immune infiltration scores (Supplementary Fig. 5D, E). In addition, TET2/3 and STAT1/3 were inversely correlated with immunosuppressive M2 macrophages (Supplementary Fig. 5F). Furthermore, we analyzed the correlation of STAT1/3 expression with that of CD274 in the transcriptome and found that the expression of both STAT1 and STAT3 was positively correlated with the expression of CD274 (Supplementary Fig. 5G), consistent with previous reports [22, 23]. Overall, based on the RNA-seq results for melanoma tumor tissues, we concluded that α-KG promotes IFNγ-STAT1/3-CD274 signaling through TET2/3, which in turn improves immune responsiveness and ultimately enhances the therapeutic efficacy of anti-PD1 therapy.

### TET2/3-STAT1/3-CD274 signaling was upregulated in tumors in mice that responded to anti-PD1 treatment

**Fig. 3:**
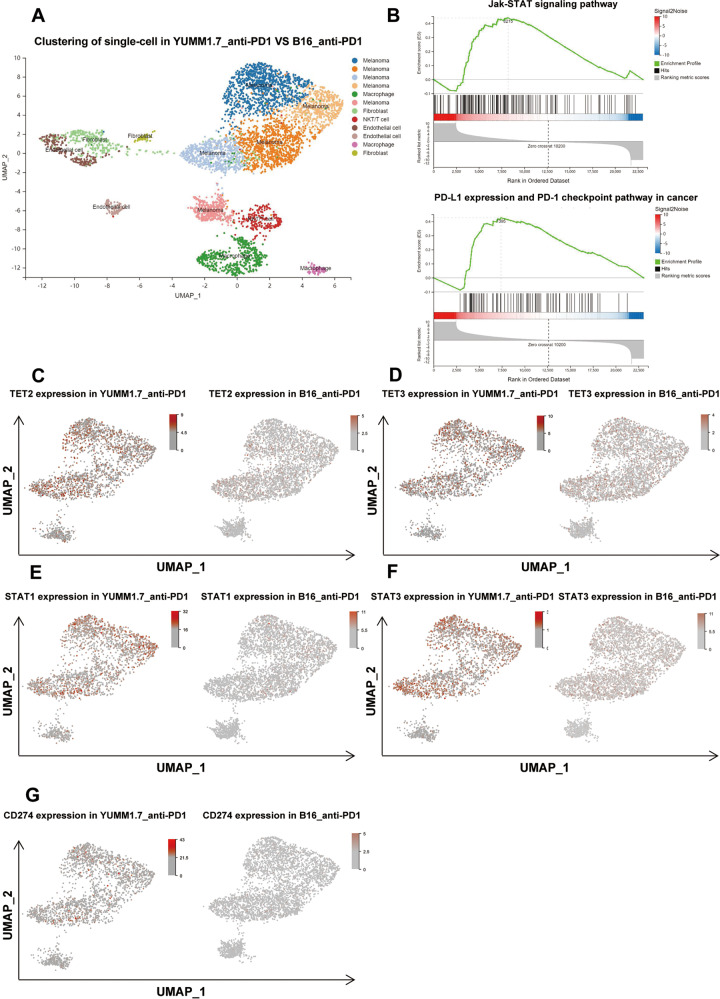
TET2/3-STAT1/3-CD274 signaling was upregulated in tumors of mice that responded to anti-PD1 treatment. **A** Plot of Uniform manifold approximation and projection (UMAP) for anti-PD1 treatment profiles (number of tumors from B16F10 tumor-bearing mice: *n* = 2 and number of tumors from Yumm1.7 tumor-bearing mice: *n* = 2; *n* = 21 516 cells), with color coding indicating cell clusters. **B** Enrichment map of GSEA signaling pathway enriched in Yumm1.7_ anti-PD1 group compared to B16F10_ anti-PD1 group. **C**–**E**, Gene expression in Yumm1.7_ anti-PD1 or B16F10_ anti-PD1 single cell clusters.

To verify the relevance of the TET2/3-STAT1/3-CD274 signaling axis to the efficacy of anti-PD1 treatment, we used single-cell sequencing to elucidate the overall landscape of B16F10 and Yumm1.7 tumors in mice after anti-PD1 mAb treatment (Fig. 3A, Supplementary Fig. 6A). Similar to the overall transcriptomic analysis results, GSEA revealed significant enrichment of the JAK-STAT and PD1-PD-L1 signaling pathways in melanoma clusters (Fig. 3B). We further found that TET2/3, STAT1/3 and CD274 were more highly expressed in anti-PD1-treated Yumm1.7-derived melanoma cells than in similarly treated B16F10 cells (Fig. 3C–G). Similarly, we used violin plots to demonstrate the gene expression levels of components of the TET2/3-STAT1/3-CD274 signaling axis in melanoma cluster (Clusters 0, 1, 2, 3 and 5) (Supplementary Fig. 6B–F). Overall, the above single-cell analysis further suggests that the TET2/3-STAT1/3-CD274 signaling axis correlates with anti-PD1 treatment sensitivity.

### The TET2/3-STAT1/3-CD274 pathway was positively correlated with anti-PD1 treatment responses in melanoma patients

Next, to validate our preclinical observations in melanoma patients, we investigated the expression of components of the TET-STAT1/3-CD274 axis in several melanoma datasets. Consistent with our above results, TET2/3, but not TET1, were downregulated in melanoma patients in multiple GEO datasets (Supplementary Fig. 7A, B), and TET3 expression was positively correlated with STAT3 expression (Supplementary Fig. 7C). Similarly, online TCGA/GTEx database analysis revealed that TET2/3 expression was significantly lower in skin cutaneous melanoma (SKCM) patients than in normal people (Supplementary Fig. 7D) and that TET expression positively correlated with that of STAT1/3 (Supplementary Fig. 7E, F) [24].

**Fig. 4:**
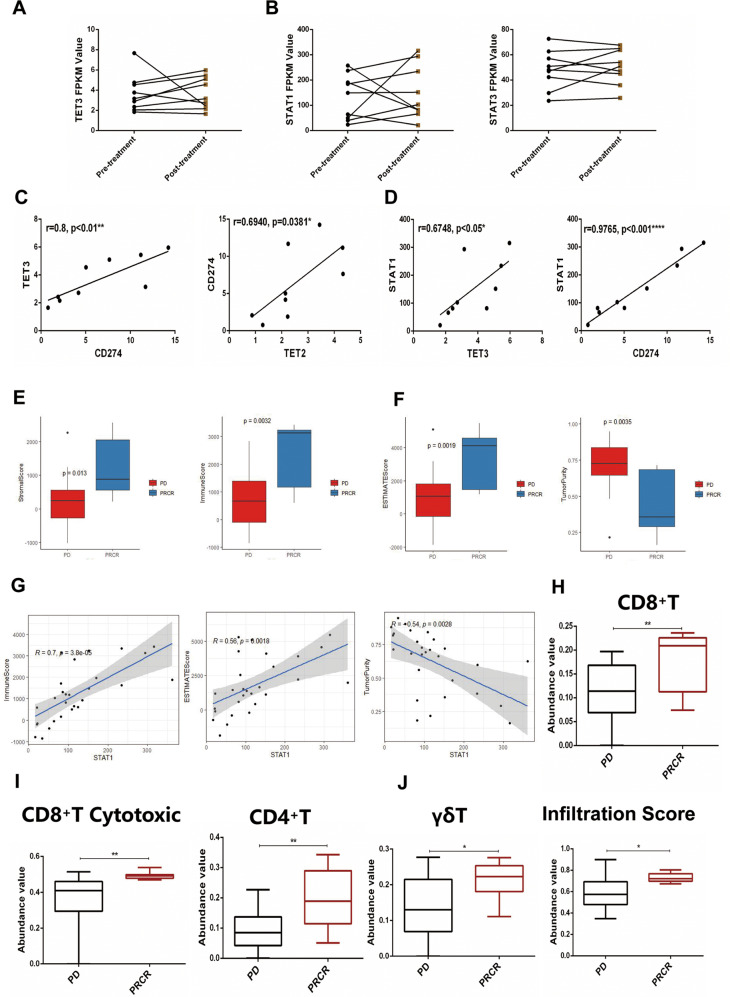
TET2/3-STAT1/3-CD274 signaling positively correlates with anti-PD1 treatment responsiveness in melanoma patients. **A**, **B** Data from the GEO database (GSE 91061) were used to analyze the TET2/3 **A** and p-STAT1/3 **B** expression in PD-1 blockade-treated melanoma patients. **C**, **D** Data from the GEO database (GSE 91061) were used to analyze the relevance of TETs and STAT-CD274 signaling. The results are expressed as the Spearman rank correlation coefficient. **E**, **F** Data from the GEO database (GSE 91061) were analyzed by the ESTIMATE algorithm to characterize the relationships between the PD or PRCR groups and immune infiltration. G, Data from the GEO database (GSE 91061) were analyzed by the ESTIMATE algorithm to characterize the correlations between STAT1 expression and immune scores (**G**, left panel), estimate scores (**G**, middle panel) or tumor purity (**G**, right panel). **H**–**J** Data from the GEO database (GSE 91061) were analyzed by the ImmuneCellAI-human method to characterize the immune microenvironment of melanoma tissue. The relative proportions of CD8^+^ T cells **H**, cytotoxic CD8^+^ T cells (**I**, left panel), CD4^+^ T cells (**I**, right panel), and γδ T cells (**J**, left panel) and immune infiltration scores (**J**, right panel) in the tumor microenvironment. Data are expressed as the mean (PD *n* = 19, PRCR *n* = 9) ± SD. PR partial response, CR complete response, PD progressive disease. Asterisks (*) indicate the degree of significant difference.

Furthermore, we evaluated the relationship between TET-STAT-CD274 signaling and immunotherapeutic efficacy in anti-PD1-treated melanoma patients from the GEO database and found that compared to the corresponding pretreatment expression, TET3 (6/9), STAT1 (5/9), and STAT3 (5/9) expression after treatment was increased in more than 55–66% of PRCR patients (Fig. 4A, B). Notably, TET-STAT-CD274 signaling molecule expression levels were highly correlated in 9 paired PRCR melanoma patients (Fig. 4C, D). Additionally, immune infiltration correlations were calculated by using the ESTIMATE algorithm. Based on the calculations, we found that the stromal score, immune score and ESTIMATE score were significantly higher in the PRCR group than in the PD group and that tumor purity was lower in the PD group, indicating that the immune microenvironment was improved in the PRCR group (Fig. 4E, F). By further analyzing the relationship between TET-STAT-CD274 signaling and the ESTIMATE score, we found that TET3 expression was positively correlated with the immune score (Supplementary Fig. 8A). STAT1 expression was highly correlated with the immune score and ESTIMATE score and negatively correlated with tumor purity (Fig. 4G). In addition, CD274 expression was positively correlated with the stromal score, immune score and ESTIMATE score and inversely correlated with tumor purity (Supplementary Fig. 8B, C). Similar to the ESTIMATE results, the immune cell composition measured by the ImmuneCellAI-human method [25] was significantly improved in the PRCR group, which had significantly higher level of CD8^+^ T cells, CD4^+^ T cells, cytotoxic CD8^+^ T cells, and γδ T cells than the PD group; the PRCR group also had better immune infiltration scores (Fig. 4H–J). Further correlation analysis demonstrated that STAT1 and CD274 expression was positively correlated with the CD8^+^ T-cell, cytotoxic CD8^+^ T-cell, and γδ T-cell expression scores (Supplementary Fig. 8D, E). Together, these analyses verify that TET2/3-STAT1/3-CD274 signaling predicts a better immune microenvironment and better responsiveness to PD1 blockade in melanoma patients.

### α-Ketoglutarate regulated the IFNγ-induced STAT1/3-PD-L1 pathway through TET2/3

**Fig. 5:**
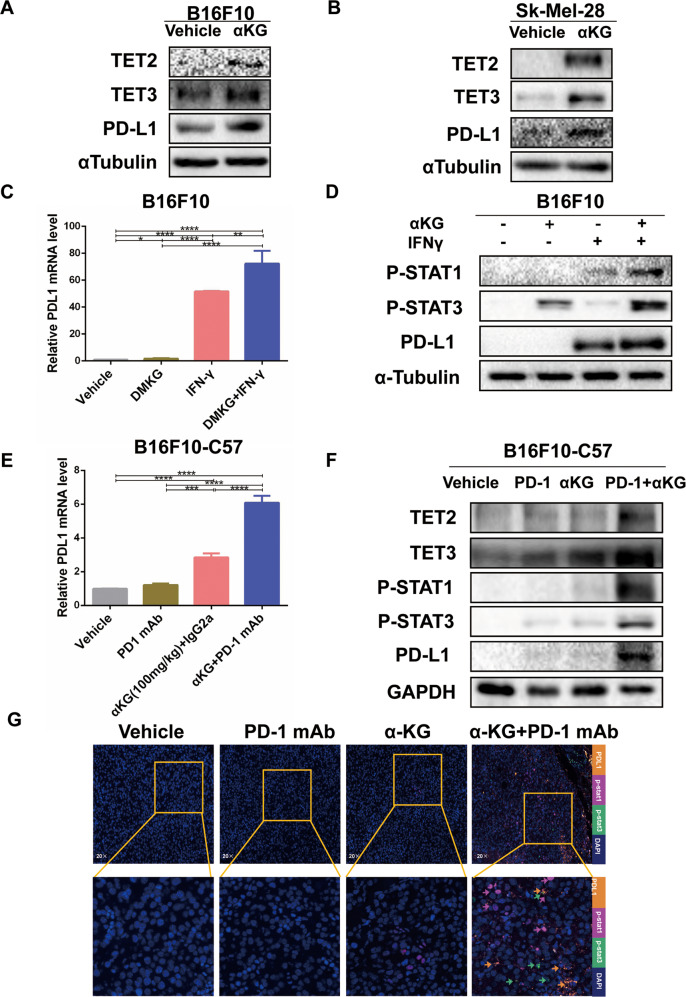
α-ketoglutarate upregulated the expression of TET2/3 and IFNγ-induced STAT1/3 and PD-L1 in melanoma cells. **A**, **B** Cell lysates were extracted from B16F10 **A** and Sk-Mel-28 **B** cells, and immunoblotting was then performed. **C**, **D** Extraction of mRNA and protein from treated melanoma cells, and qRT–PCR **C** and immunoblotting **D** were then performed. **E**, **F** Extraction of mRNA and protein from treated B16F10 tumor-bearing mouse tissues, and qRT-PCR **E** and immunoblotting **F** were then performed. **G** Detection of the expression of p-STAT1/3 and PD-L1 in B16F10 tumor-bearing mouse tissues by TSA-fluorescent multiplex immunofluorescence staining after the indicated treatments. Multiple experimental data (*n* = 3) are counted and presented according to the statistical methods, and an asterisk (*) indicates the degree of significant difference.

To further corroborate the link between α-KG-TET2/3 and PD-L1, we treated melanoma cells with dm-α-KG, and the results showed that α-KG promoted the protein expression of PD-L1 and TET2/3 in melanoma cells (Fig. 5A, B). Moreover, α-KG significantly elevated the expression of PD-L1 induced by IFNγ at both the mRNA and protein levels (Fig. 5C, D, Supplementary Fig. 9A, B). In addition, α-KG increased the IFNγ-induced expression of p-STAT1/3 (Fig. 5D, Supplementary Fig. 9B). Similar to the in vitro findings, α-KG supplementation led to marked PD-L1 upregulation at the RNA and protein expression levels in tumor tissue in the α-KG + anti-PD1 group (Fig. 5E, F). The expression of TET2/3 and p-STAT1/3 was also increased in tumor tissue in the α-KG + anti-PD1 group (Fig. 5F). TSA-multicolor tissue immunofluorescence further verified that p-STAT1/3-PD-L1 signaling was significantly increased in the α-KG + anti-PD1 group (Fig. 5G). These findings indicate that α-KG affects IFNγ-STAT1/3-CD274 signaling through TET2/3.

**Fig. 6:**
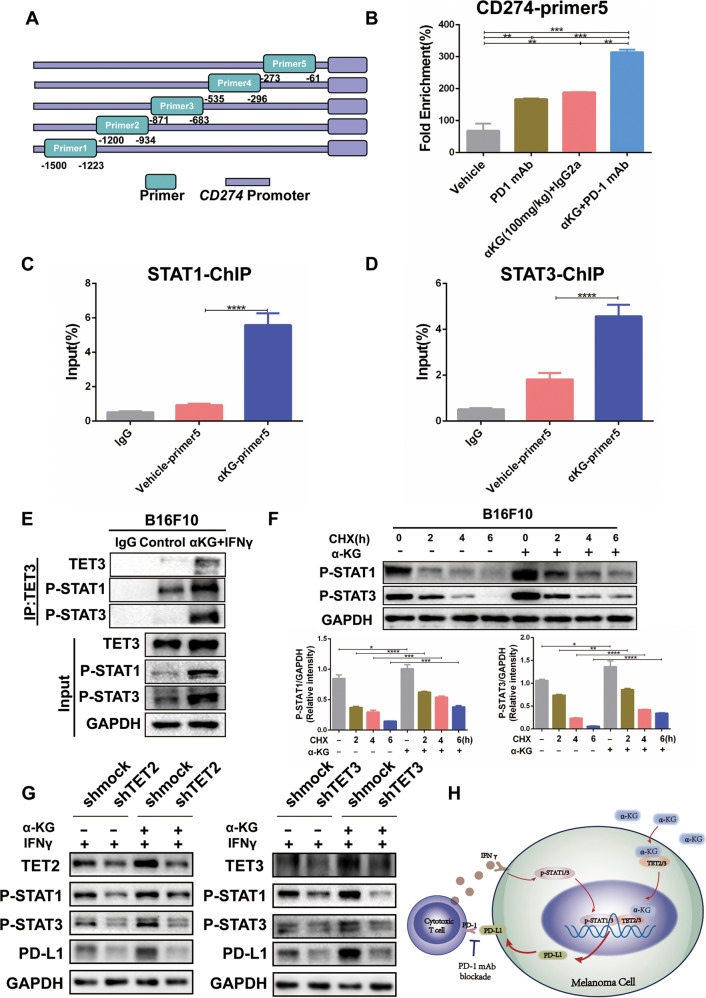
Regulation of PD-L1 by the α-KG-TET2/3-p-STAT1/3 axis is required. **A** Designed primers for hMeDIP analysis and ChIP experiments. **B** 5-hmC levels of PD-L1 promoters in B16F10 tumor-bearing mouse tissues after the designated treatments. **C**, **D** ChIP-qPCR was performed to detect the binding of STAT1 **C** or STAT3 **D** to PD-L1 promoter sites in B16F10 cells with or without α-KG treatment. **E** Immunoprecipitation analysis of TET3 with p-STAT1/3 in B16F10 cells receiving the designated treatments. **F** P-STAT1/3 protein levels at different time points after IFNγ stimulation in B16F10 cells treated with CHX (100 mg/mL) or α-KG (200 μm). **G** The expression of p-STAT1/3 and PD-L1 protein levels was detected by immunoblotting after knocking down TET2 (left panel) /TET3 (right panel) under the designated treatment. **H** Schematic diagram of the mechanism. Multiple experimental data (*n* = 3) are counted and presented according to the statistical methods, and an asterisk (*) indicates the degree of significant difference.

To further explore how α-KG-regulated TET2/3 control IFNγ-STAT1/3-CD274 signaling, five primer pairs were designed based on the −1500 bp~−1 bp region of the CD274 promoter region sequence (Fig. 6A), and the level of CD274 promoter hydroxymethylation in tumors was detected using the hydroxymethylated DNA immunoprecipitation (hMeDIP) method. Based on the results, we found that the 5-hmC levels of the CD274 promoter region (−535~−296 bp and −273~−61 bp) were significantly elevated in the α-KG + anti-PD1 group, suggesting that α-KG supplementation promotes PD-L1 expression by regulating its demethylation in response to anti-PD1 mAb treatment (Fig. 6B, Supplementary Fig. 9C). ChIP experiments further confirmed that both STAT1 and STAT3 could directly bind to the CD274 promoter and that the binding of STAT1/3 to the CD274 promoter was enhanced by the addition of α-KG (Fig. 6C, D, Supplementary Fig. 9D). In addition, since both TET2/3 and STAT1/3 are capable of binding to the PD-L1 promoter, we evaluated whether there is an interaction between them. Specifically, we examined whether there is a direct interaction between TET2/3 and STAT1/3 and found that TET2/3 could directly bind to p-STAT1/3 and that the addition of α-KG and IFNγ enhanced the interaction (Fig. 6E, Supplementary Fig. 9E). Furthermore, α-KG significantly decreased the degradation of IFNγ-induced p-STAT1/3 after treatment with the protein synthesis inhibitor cycloheximide (CHX) (Fig. 6F, Supplementary Fig. 9F). Moreover, the enhancement of IFNγ-STAT1/3-CD274 signaling by α-KG was diminished after inhibition of TET2/3 (Fig. 6G). Taken together, these findings indicate that α-KG-activated TET2/3 bind to the CD274 locus to promote CD274 demethylation and transcription.

## Discussion

PD-L1, also named CD274, is a typical transmembrane immunoglobulin protein that functions in association with PD1 in the restriction of immune cell hyperactivation, including CD8^+^ T-cell hyperactivation, and maintenance of immune homeostasis [26]. However, under tumorigenic conditions, tumor cells express PD-L1 to trigger the PD-L1/PD1 signaling pathway in T cells and block CD8^+^ T-cell activation, leading to tumor immune escape [23]. Anti-PD1/PD-L1 treatment blocks the interaction between PD-L1 and PD1 and activates CD8^+^ T cells to kill cancer cells, which produces a promising antitumor effect; therefore, PD-L1 expression has been considered to be a predictive biomarker for response to anti-PD1 therapy [27].

Metabolic reprogramming is one of the hallmarks of tumors, and it not only plays a crucial role in tumorigenesis and tumor signaling but also has a broader significance in antitumor effects through the release of metabolism-related factors (e.g., lactate, arginine, tryptophan, IFNγ, and PD-L1) [1, 28]. For example, in melanoma patients, the LDHA and lactate levels in tumors are negatively correlated with antitumor CD8^+^ T-cell activity and overall patient survival [29]. Lactate is exported to the extracellular environment through monocarboxylate transporter (MCT), which creates an acidic tumor microenvironment (TME) [30] that results in aggressive tumor cells and suppression of antitumor immunity [31]. Our previous studies also showed that dysregulated glutamate-cystine transport in melanoma cells promoted the secretion of exosomal PD-L1, which in turn induced a shift in macrophages toward the M2 phenotype, ultimately leading to an inhibitory tumor microenvironment [13].

Emerging evidence has demonstrated that combination treatment involving metabolic pathway targeting and immune checkpoint inhibitor treatment is a promising antitumor therapeutic strategy [32]. Phosphofructokinase 2/fructose 2,6-bisphosphatase 3 performs crucial roles in glycolysis and lactate production in tumor cells [33], and the combination inhibitor PFK-158 targets PFKFB3 with cytotoxic T lymphocyte antigen 4 and enhances therapeutic efficacy in a melanoma model [34]. In addition, arginine supplementation stimulates cytotoxicity and effector cytokine production in T and NK cells in vitro and, when combined with anti-PD-L1 treatment, significantly enhances antitumor activity and prolongs survival in osteosarcoma [35]. Inhibition of Arg-1 and iNOS expression inhibits polyamine production and the TCA cycle, thereby further suppressing the immunosuppressive capacity of PMN-MDSCs and enhancing the antitumor effects of anti-PD1 mAb therapy in B16-F10 and 4 T1 mouse tumor models [36]. Combined interventions comprising metabolic and immune-based therapies are currently being tested in several clinical trials, including an ongoing clinical trial evaluating the ARG1 inhibitor INCB001158 in combination with the ICI pembrolizumab for the treatment of solid tumors (NCT02903914) [32].

In this research, we revealed that the α-KG plasma level was dramatically reduced in melanoma patients compared with healthy individuals (Supplementary Fig. 1A). Furthermore, we found that although α-KG did not affect melanoma cell proliferation or clonogenic ability (Supplementary Fig. 1), supplementation with α-KG significantly enhanced the efficacy of an anti-PD1 mAb in melanoma (Fig. 1). However, the role of α-KG in tumorigenesis is controversial; for example, in p53-deficient pancreatic ductal adenocarcinoma (PDAC), α-KG is involved in P53-mediated tumor suppression by specifically upregulating 5-hydroxymethylcytosine (5 hmC) to elevate tumor suppressor gene expression [37]. In breast cancer, α-ketoglutarate activates collagen hydroxylation by increasing collagen prolyl-4-hydroxylase (P4HA) activity, which impairs the growth of breast cancer-derived lung metastases [38]. In contrast, in esophageal squamous cell carcinoma (ESCC), isocitrate dehydrogenase 3beta (IDH3beta) facilitates tumor cell proliferation by increasing α-KG production [39].

Accumulating evidence has shown that α-KG acts not only as an intermediate product of the TCA cycle but also as a signaling molecule in response to DNA posttranslational modifications and oxidative homeostasis [40, 41]. α-KG is an essential cosubstrate for 2-oxoglutarate-dependent dioxygenases, which regulate the enzymatic activity of Ten-Eleven-Translocation (TET) enzymes, Jumonji-domain-containing family histone demethylases and prolyl-4 hydroxylase [41, 42]. α-KG hinders embryonic stem cell (ESC) differentiation by regulating 5-hydroxymethylcytosine and histone H3K9 trimethylation levels in the regulatory regions of core transcription factors [4]. α-KG also binds to and blocks the activity of ATP synthase subunit β, leading to a reduced ATP content and oxygen consumption and increased autophagy, which prolongs the lifespan of adult *Cryptobacterium hidradi* [43].

To elucidate how α-KG enhances the efficacy of anti-PD1 immunotherapy in melanoma, we conducted RNA sequencing of tumors treated with α-KG, anti-PD1 or α-KG + anti-PD1 and found that IFNG-STAT1/3-CD274 signaling and α-KG-dependent TET2/3 were significantly altered in the α-KG + anti-PD1 mAb group. Moreover, α-KG treatment dramatically increased the expression of TET2/3 (Fig. 5A, B) but did not affect histone demethylation (Supplementary Fig. 5A, B). Furthermore, we confirmed that α-KG enhanced IFNγ-induced PD-L1 expression and p-STAT1/3 protein expression (Fig. 5C–G; Supplementary Fig. S9A, B). More importantly, α-KG treatment elevated the 5-hmC level and stabilized p-STAT1/3 binding in the PD-L1 promoter (Fig. 6), suggesting that α-KG plays a pivotal role in the IFNγ-STAT1/3-CD274 axis through TET2/3 producing 5-hmC in the PD-L1 promoter.

Previous findings have shown that tumor cells increase PD-L1 expression through transcription factors such as IRF1/4, c-Myc, EGR1, c-Jun, HIF-1α, NF-κB, ATF3 and STAT3 [13, 23, 44]. In our study, we showed that not only STAT3 [23, 45] but also STAT1 could directly bind to the PD-L1 promoter and regulate PD-L1 expression (Fig. 6C), which is a further refinement of the PD-L1 regulatory mechanism.

Growing evidence has shown that supplementation with α-KG produces benefits in clinical and experimental nutrition [40]. α-KG improves myocardial protection during cardiac surgery by reducing metabolic abnormalities and mitigating ischemic injury [46, 47]. Kirkman Corporation in the United States sells α-KG as a dietary supplement, while Double Wood, Inc. sells α-KG as a dietary supplement to support athletic performance, which demonstrates the safety and application potential of α-KG. Moreover, α-KG has a low extraction cost and good stability when dissolved in water [48], which has great potential for clinical applications. In summary, our findings confirmed that α-KG enhanced the immunotherapeutic efficacy of anti-PD1 through the TET2/3-STAT1/3-CD274 pathway and that supplementation with α-KG plus treatment with anti-PD1 might be a novel strategy for melanoma treatment.

Overall, α-KG significantly increased the level of the methylcytosine dioxygenases TET2/3, which led to an increased 5-hydroxymethylcytosine (5-hmC) level in the PD-L1 promoter. As a consequence, STAT1/3 was recognized and stabilized in the PD-L1 promoter to upregulate PD-L1 expression (Fig. 6H). Thus, we provide novel insight into the role of α-KG in melanoma and the associated epigenetic mechanism to improve the efficacy of anti-PD1 therapy.

## Materials and methods

### Plasma collection and nuclear magnetic resonance spectroscopy (NMR) analysis in melanoma patients

Central South University Xiangya Hospital Ethics Committee approved the human study and it was conducted in compliance with the Declaration of Helsinki. All patients provided written informed consent (ethic code: 201803363). We then collected plasma samples from melanoma (*n* = 20) and normal subjects (*n* = 20) and analyzed them according to the previous manipulations [13].

### Cell culture

B16F10 and Yumm1.7 mouse malignant melanoma cells and Sk-Mel-28 human malignant melanoma cells were obtained from American Type Culture Collection (ATCC; USA). B16F10 was cultured in RPMI 1640 medium (BI, Israel) containing 10% fetal bovine serum (FBS; Gibco, USA). Yumm1.7 was cultured in DMEM/F-12 (HyClone, USA) with 10% FBS (Gibco, USA) and 100× MEM NEAA (Gibco, USA). Cultured Sk-Mel-28 at 37 °C and 5% CO2 in DMEM (BI, Israel) with 10% FBS (Gibco, USA). All cell lines have been authenticated using STR profiling.

### α-ketoglutarate assay

To initially observe the relationship between α-KG and anti-PD1 treatment, plasma samples from patients with melanoma treated with anti-PD1 as adjuvant therapy after surgery were collected and tested by ELISA. The study protocol was approved by the Clinical Committee of Xiangya Hospital. Blood samples were collected according to the informed consent policy (approval number: 2022020130). Evaluation criteria were referenced to studies [44, 49, 50]. Patients were stratified into response groups based on RECIST 1.1 criteria. Patients with non-recurrence > 3 months were classified as responders and SD (stable disease), while patients with SD ≤ 3 months were classified as non-responders and PD (progressive disease). SD, stable disease; PD, progressive disease. TNM stage based on the 8th Edition AJCC Cancer Stage Classification. Supplementary Tables S2 summarize the clinical information. α-Ketoglutarate was detected following instructions using an assay kit (Sigma-Aldrich, USA).

### Immunoblotting

Melanoma cells or tumor tissues were lysed in a radioimmunoprecipitation assay buffer containing protease and phosphatase inhibitors (CWBIO, China). BCA assay kit (CWBIO, China) was used to determine the Melanoma protein concentrations. On 8% or 10% SDS-polyacrylamide gel electrophoresis gels, proteins were loaded and then transferred to polyvinylidene fluoride membranes (Millipore, USA) for Western blot analysis. The following antibodies were used: TET2 (CST, 45010#, USA), TET3 (CST, 99980#, USA), p-STAT1 (CST, 9167#, USA), p-STAT3 (CST, 9145#, USA), PD-L1 (Mouse) (Abcam, ab213480, USA), PD-L1 (Human) (Abcam, ab213524, USA), α-tubulin (Proteintech, 11224-1-AP, China) and GAPDH (Proteintech, 60004-1-Ig, China). We analyzed the gel images with a gel image analysis system (Bio-Rad, USA).

### Analyses of qRT-PCR

Total RNA was isolated from cells or tissues with TRIpure Reagent (Bio Teke, China) and cDNA was synthesized with cDNA synthesis reagent (Yeasen, China). qPCR analysis was performed with qPCR Mix (Bimake, China) on a RT-PCR-Q3 system (Applied Biosystems, USA) for qPCR analysis. PCR primers are listed in Supplementary Table 1.

### Melanoma animal models

Xiangya Hospital (Central South University, China) Ethics Committee approved the animal studies and it was conducted in compliance with the “3 R” principle. For the tumor immunotherapy model, B16F10 (100 µl RPMI 1640 containing 5 × 10^5^ cells) or Yumm1.7 (100 µl DME/F-12 medium containing 5 × 10^5^ cells) cells were collected and injected into the right abdomen of female C57BL/6 mice (7–8-week-old) (SLAC Laboratory Animal Co., Ltd., Shanghai, China). When the tumor volume reached approximately 50 cubic millimeters, the tumors were randomly grouped (six in each group) and injected intraperitoneally with corn oil (vehicle) (Aladdin, China), 100 mg/kg α-KG (Sigma-Aldrich, USA), IgG2a (BioXCell, BE0089, USA) or PD1 mAb (BioXCell, BE0146, USA) for 9–11 days. Tumor volume was calculated using the formula V = (length × width^2^)/2. When the tumor reached approximately 1000 mm^3^, tumor and blood samples were collected for subsequent analysis. The investigator was blinded to the group allocation of the animals during the experiment. The sample size of each experiment is shown in the legend. No data were excluded from the analysis.

### Fluorescence-activated cell sorting (FACS) analysis

Melanoma tumor tissues were prepared as single cell suspensions, blocked with anti-CD16/CD32, and the dead cells were removed with a Zombie Red Fixable Viability Kit. Single cells were stained using APCCY7-CD45 antibody (BioLegend, CAT#:103116, USA), APC-CD3 antibody (BioLegend, CAT#:100236, USA), PE-Cy5.5-CD4 antibody (BioLegend, CAT#:100434 USA), PE-Cy7-CD8 antibody (BioLegend, CAT#:100722, USA), PE/Dazzle 594-CD274 antibody (BioLegend, CAT#:124324, USA), APC-F4/80 antibody (BioLegend, CAT#:123116, USA) and PE-CD11B antibody (BioLegend, CAT#:101208, USA) for 30 min. After fixation and permeabilization (eBioscience, USA), intracellular staining was performed using FITC-Granzyme B antibody (BioLegend, CAT#:372206, USA), BV711-IFNγ antibody (BioLegend, CAT#:505836, USA) and PE-Cy7-CD206 antibody (BioLegend, CAT#:141720, USA). The antibodies used in animal flow cytometry are from biolegend, Inc. The stained cells were analyzed by FACS (Cytek, USA) and Flow Jo software.

### RNA sequencing

Tumor tissues were collected from the vehicle, α-KG, anti-PD1 mAb and α-KG + anti-PD1 groups of mice, with four independent tumor tissue samples collected from each group. Total RNA extraction, cDNA library construction, library purification and transcriptome sequencing of melanoma tissues were performed by Wuhan Huada Sequencing Company following the manufacturer’s instruction.

### Single cell sequencing and analysis

The tumors from B16F10_anti-PD1 and Yumm1.7_anti-PD1 were prepared as single cell suspensions for counting, viability and concentration detection by Scanner. The single cell suspensions were injected into Cartridge for cell sedimentation. Subsequent capture of mRNA and antibody tags is performed using Poly-dT on magnetic beads. A cDNA library is constructed from the mRNA on the magnetic beads. At the same time, library construction is performed on Sample Tag on magnetic beads. Data comparison and data statistics were performed by matching and splitting the CL and UMI sequences on the two library sequences. Subsequently, BD genomics rhapsody was used for scRNA-Seq gene expression quantification based on UMI counts. Seurat software was used for data quality control and filtering: the number of genes identified in the cells was filtered out from less than 200 or greater than 90% of the maximum number of genes; the top 15% of mitochondrial reads were filtered out; and the effect of cell cycle was corrected; Seurat and Doubletdetection software were used for high variable features screening and subsequent dimensionality reduction analysis, multiplex data integration, difference analysis, pathway enrichment, etc.

### Hydroxymethylated DNA immunoprecipitation (hMeDIP) analysis

For the detection of the 5-hmc level in the CD274 promoter, DNA was isolated from tumor tissues. A DNA hydroxymethylation immunoprecipitation kit (A&D Technology, China) was used to detect DNA hydroxymethylation according to instructions.

### Immunoprecipitation

Melanoma cells were lysed in NP-40 lysis buffer (Beyotime, China) containing protease and phosphatase inhibitors (Selleck, USA). To block nonspecific protein binding, the cell lysates were first incubated with a normal IgG antibody (CST, 2729#, USA) or protein A/G-agarose (Beyotime, China) for 1 hour at 4 °C. The beads were discarded, and the supernatant was retained. The supernatant was incubated with a normal IgG antibody or anti-TET3 (1:1 000, CST) and protein A/G-agarose (Beyotime, China) for 4 h at 4 °C. Then, the beads were washed 3 times with NP-40 buffer and loaded to 8% or 10% SDS–PAGE for immunoblotting.

### ChIP–qPCR assay

The ChIP assay was performed using a SimpleChIP® Enzymatic Chromatin IP Kit (CST, USA) following the manufacturer’s instruction. ChIP-enriched DNA was analyzed by qPCR Mix (Bimake, China) on a RT-PCR-Q3 system (Applied Biosystems, USA) for qPCR analysis. PCR primers are listed in Supplementary Table 1.

### TSA-multiplex immunofluorescence staining

We used an Opal 7-color fluorescent IHC kit (MEL797001KT, PerkinElmer Inc., USA) for multiplex immunofluorescence staining. Formalin-fixed, paraffin-embedded tumor sections were first incubated with primary antibody (PD-L1 antibody, 1:500 dilution; CST, USA), followed by secondary antibody and Opal-620 working solution after antigen retrieval and blocking. Sections were then subjected to antigen retrieval and antibody stripping by microwave heating. Next, the process was repeated using a second antibody (p-STAT1 antibody, 1:1000 dilution; CST, USA) and Opal-650 working solution and a third antibody (p-STAT3 antibody, 1:1000 dilution; CST, USA) with Opal-570 working solution. Finally, the nuclei were stained with DAPI and the images were analyzed with a multicolor fluorescence microscope (PerkinElmer Inc., USA).

### Statistical analysis methods

Data were analyzed by unpaired Student’s t-test and one- or two-way ANOVA tests using GraphPad Prism (version 6.01). Repeated data are expressed as mean ± standard deviation (SD). *p* < 0.05 indicates a significant difference. Asterisks (*) indicate the degree of significant difference (*P < 0.05, **P < 0.01, ***P < 0.001, and ****P < 0.0001).

## Supplementary information

Supplementary figures and figure legends

Original western blots

aj-checklist

## Data Availability

RNA sequencing data are available from NCBI (GSE223110); All datasets analyzed during the current study are available from the corresponding author on reasonable request.
