# Supplementary material for: Supplementation with α-ketoglutarate improved the efficacy of anti-PD1 melanoma treatment through epigenetic modulation of PD-L1
Source: Cell Death Dis. 2023 Feb 28;14(2):170. doi: 10.1038/s41419-023-05692-5 (PMC9974984; doi:10.1038/s41419-023-05692-5)
Supplement: Supplementary file 1 — Supplementary figures and figure legends [file 41419_2023_5692_MOESM1_ESM.docx]

**Supplementary Figures and Figure legends:**

**Supplementary Figures**

**
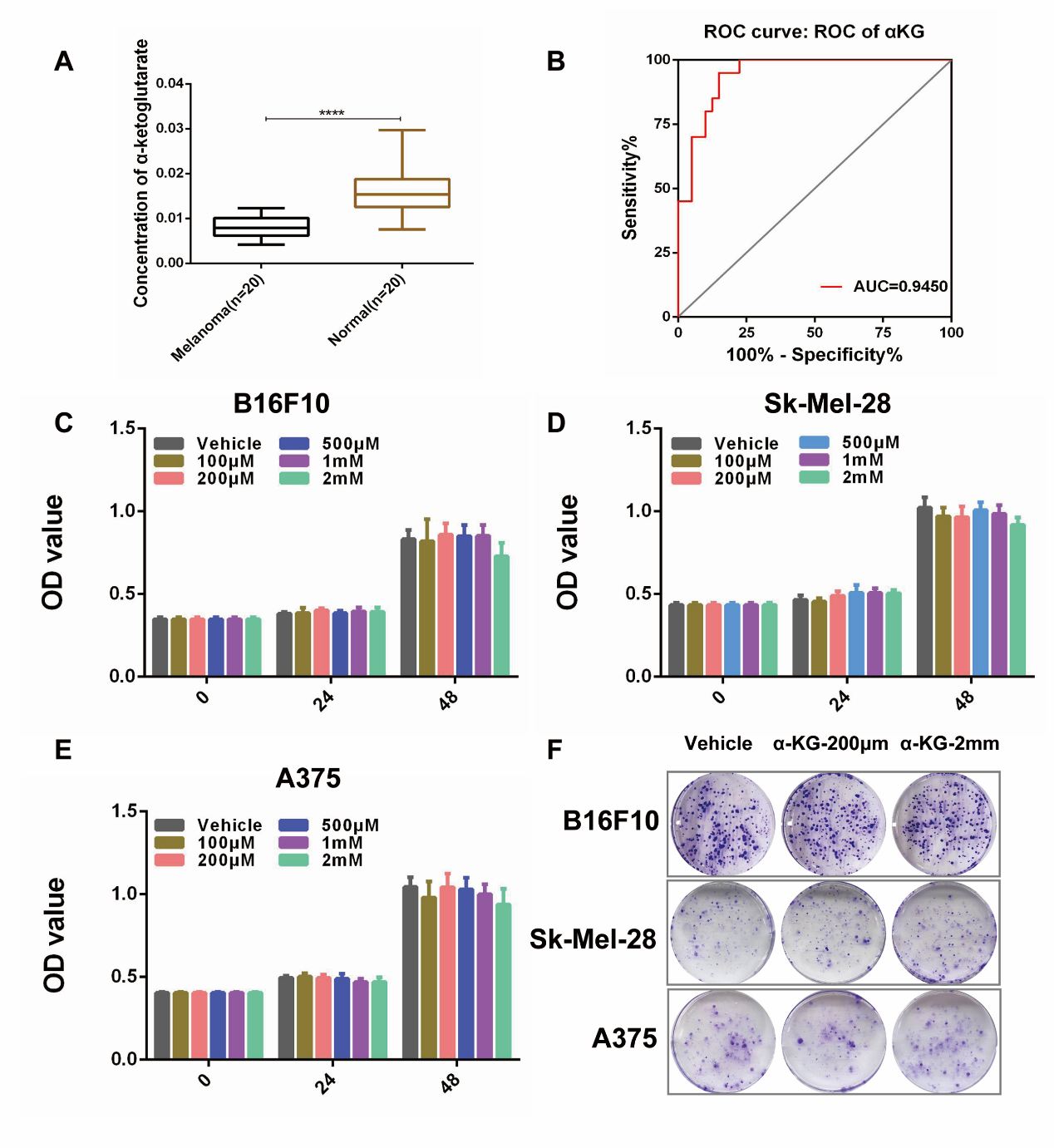
**

**Supplementary Figure 1.** **Expression level of α-ketoglutarate in melanoma patients and its effect on melanoma cells.** A, The α-KG levels detected in melanoma patient plasma (n=20) and normal subject plasma (n=20). B, The ROC curve for α-KG. C-E, B16F10, Sk-Mel-28 and A375 melanoma cells were treated with (0-2mM) α-KG for 0-48 hours, and cell viability was tested with CCK-8. F, Photographs of colony formation from B16F10, Sk-Mel-28 and A375 melanoma cells receiving the indicated treatments.


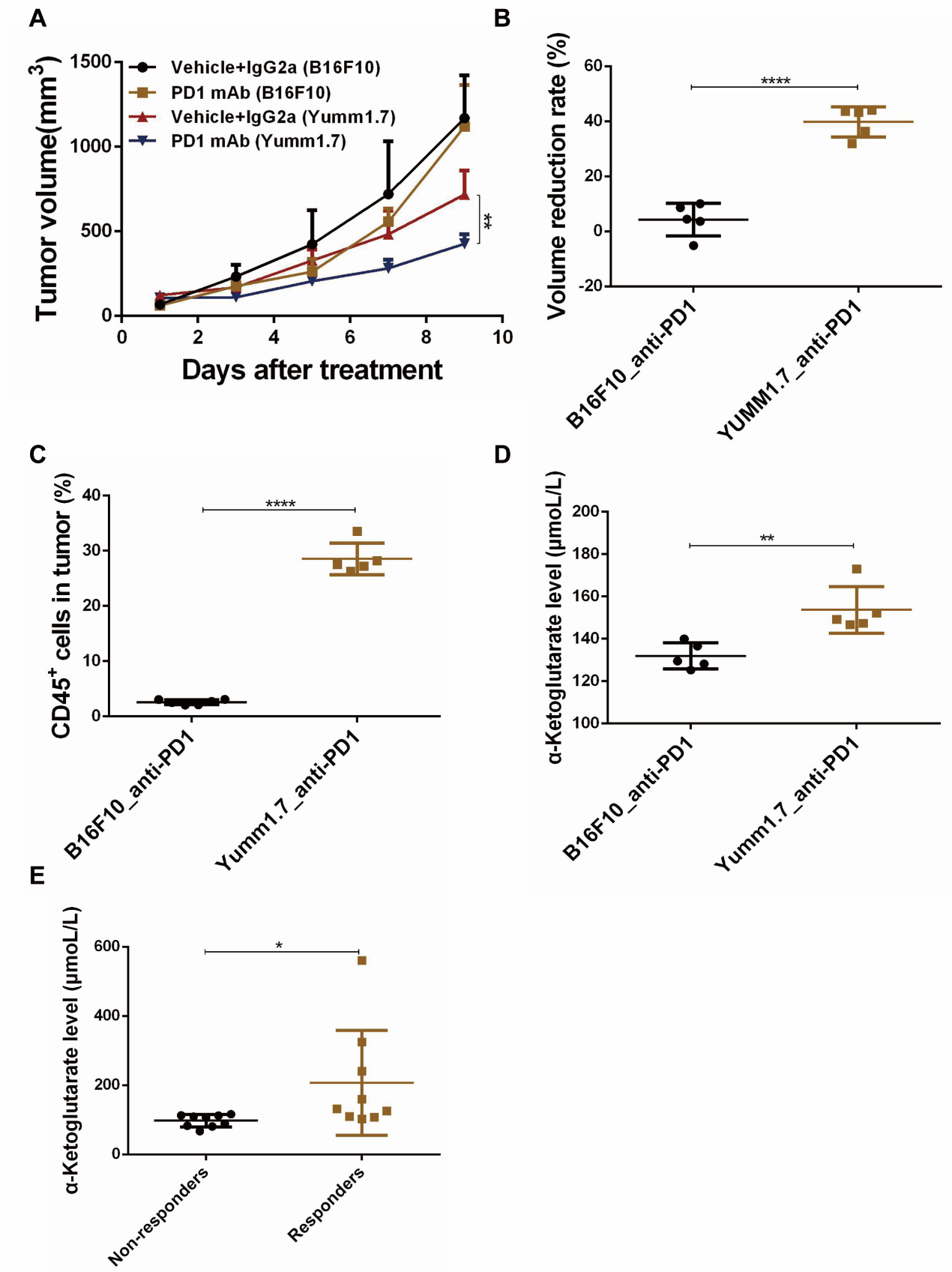


**Supplementary Figure 2. The abundance of α-ketoglutarate was related to the efficacy of PD1 mAb treatment.** A, Tumor growth curves for Yumm1.7/B16F10 tumor-bearing mice receiving the designated treatments B, Proportion of tumor reduction in B16 F10 and Yumm1.7 tumor-bearing mice after anti-PD1 treatment. C, The proportions of CD45^+^ cells were analyzed by FACS in tumors after the indicated treatments. D, α-KG levels in the serum of B16F10 tumor-bearing mice(n=5) and Yumm1.7 tumor-bearing mice(n=5) receiving anti-PD1 blockade. F, α-KG levels in the plasma of anti-PD1 non-responders (n=9) and anti-PD1 responders (n=9) receiving anti-PD1 blockade. Multiple experimental data are counted and presented according to the statistical methods, and an asterisk (*) indicates the degree of significant difference.

**
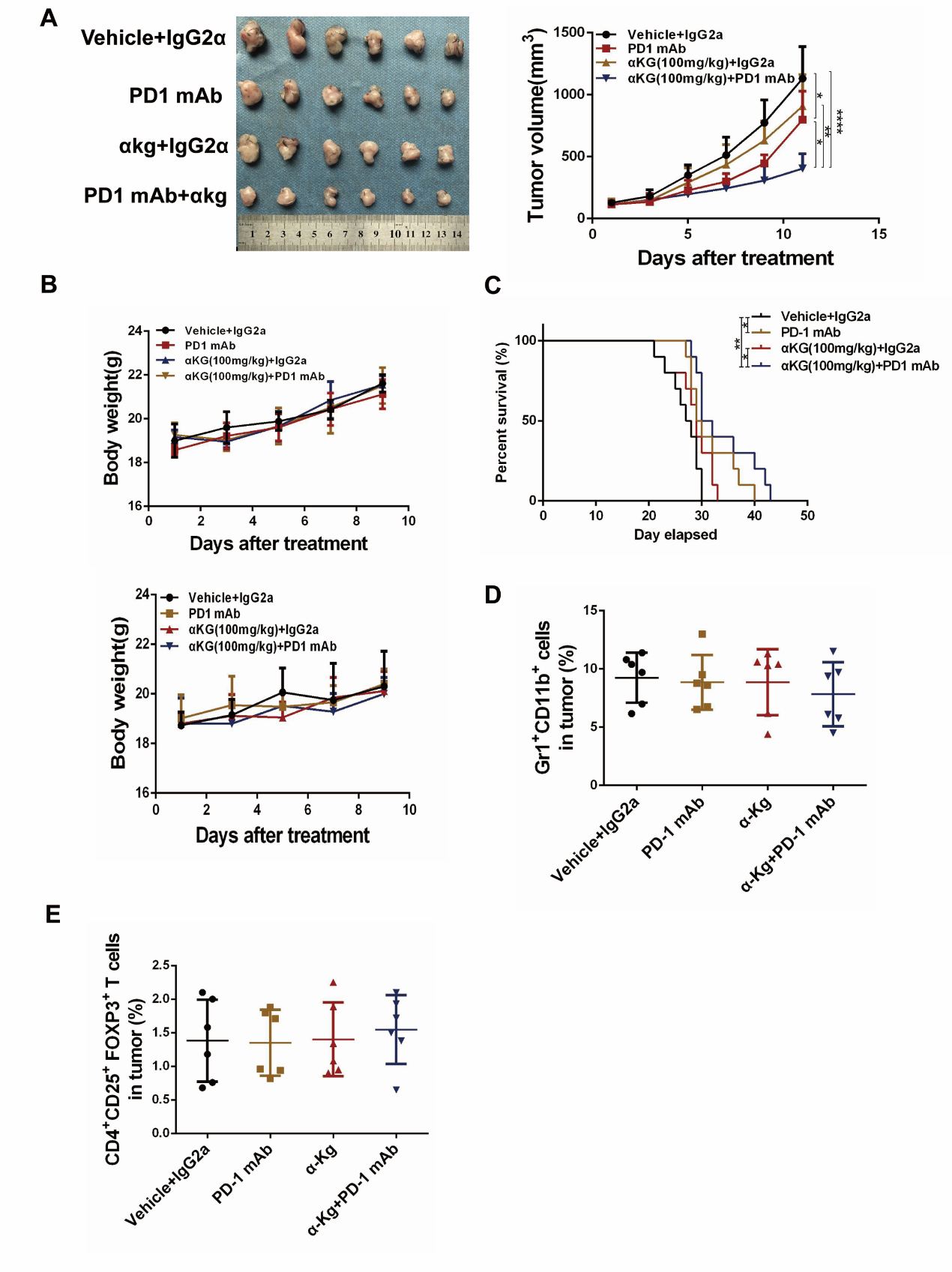
**

**Supplementary Figure 3. α-ketoglutarate enhanced the efficacy of PD-1 blockade in Yumm1.7 tumor-bearing mice.** A, Tumors isolated from Yumm1.7 tumor-bearing mice receiving the designated treatments (left panel). Tumor growth curves for Yumm1.7 tumor-bearing mice receiving the designated treatments (right panel). B, The body weight of B16F10 tumor-bearing mice (Upper Panel) and Yumm1.7 tumor-bearing mice (Lower Panel) receiving the designated treatments. C, Survival curves of Yumm1.7 tumor-bearing C57BL/6 mice. D, The proportions of Gr1^+^CD11b^+^ MDSC cells were analyzed by FACS in tumors after the indicated treatments. E, The proportions of CD4^+^CD25^+^FOXP3^+^ T cells were analyzed by FACS in tumors. Multiple experimental data (n=6) are counted and presented according to the statistical methods, and an asterisk (*) indicates the degree of significant difference.

**
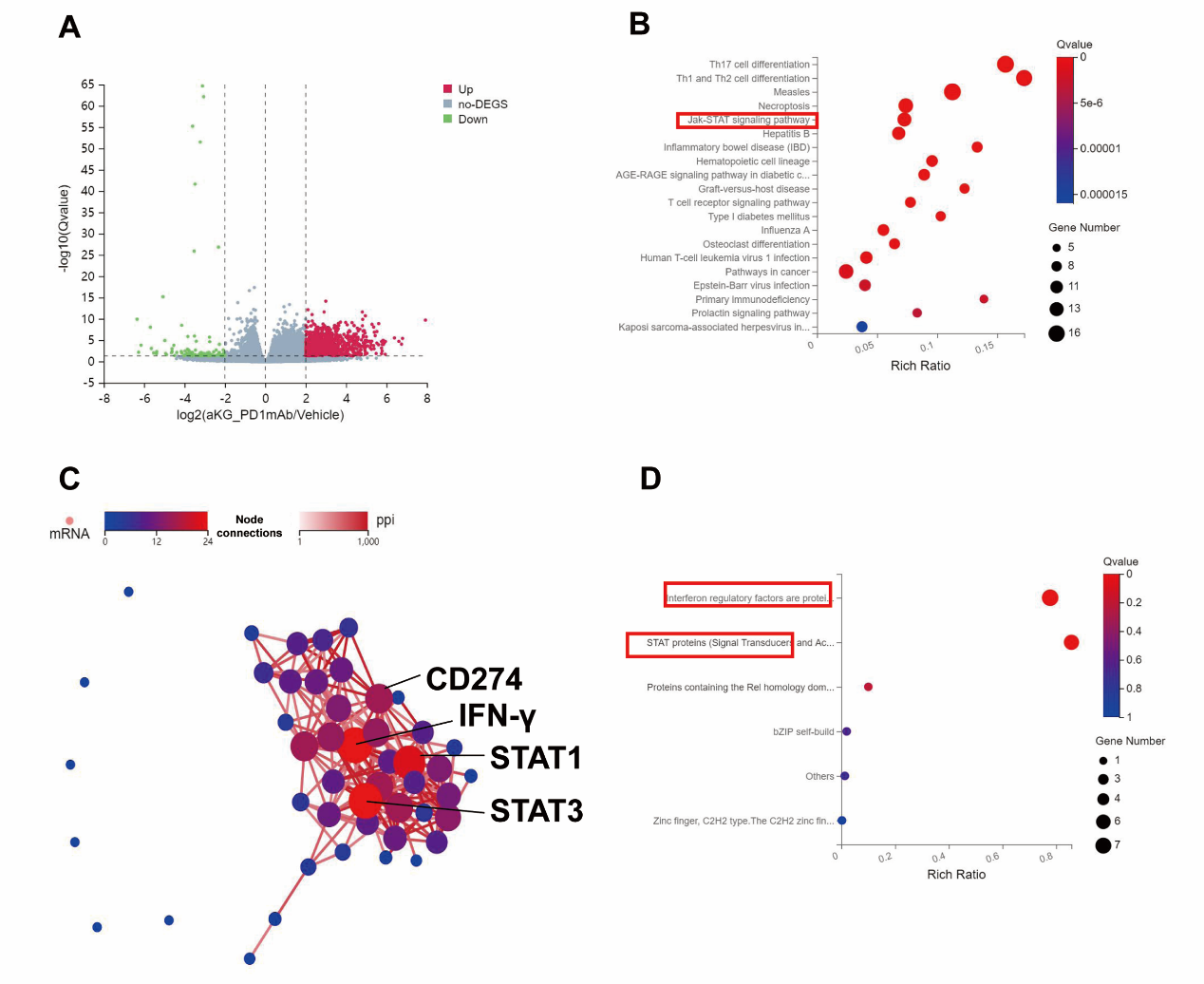
**

**Supplementary Figure 4.** **RNA-seq analyses of the effect of α-ketoglutarate and PD1 mAb treatment on the gene expression profile.** A, Differential gene volcano plot of vehicle vs α-KG+anti-PD1 groups. B, The top 20 positively enriched KEGG pathways are shown in the bubble chart for the representative differential genes. C, Protein-protein interaction (PPI) analysis was applied to analyze the interactions associated with representative differential genes. D, Transcription factor analysis was used to analyze the top 6 positively enriched transcription factor pathways associated with representative differential genes.

**
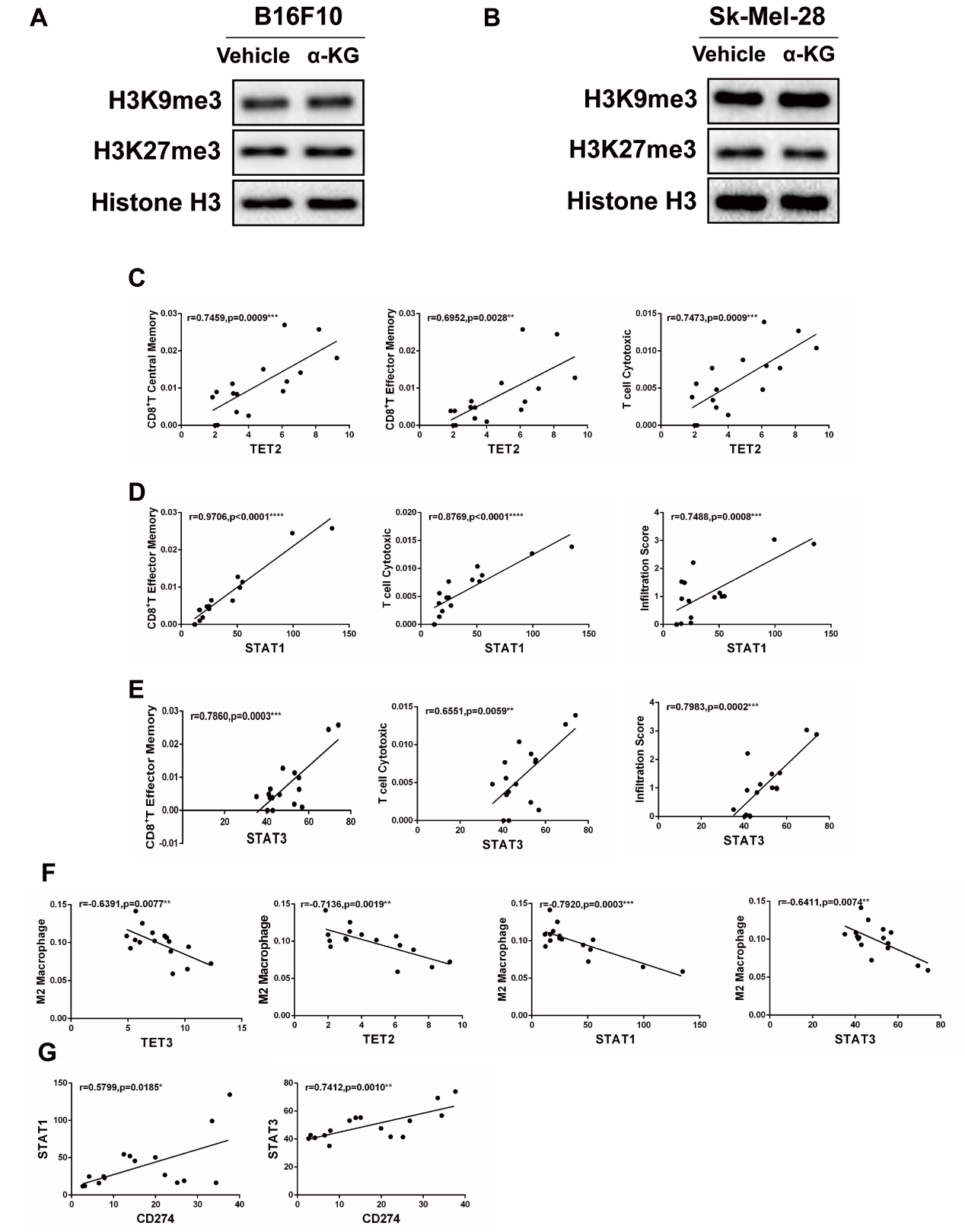
**

**Supplementary Figure 5. The correlation between TET2/3 and STAT-CD274 signaling.** A-B, Cell lysates were extracted from B16F10 (A) and Sk-Mel-28 (B) melanoma cells, and immunoblotting was then performed. C, Correlations of TET2 gene expression with the proportion of central memory CD8^+^ T cells (C, left panel), effector memory CD8^+^ T cells (C, middle panel), cytotoxic CD8^+^ T cells (C, right panel). D, Correlations of STAT1 gene expression with the proportion of effector memory CD8^+^ T cells (D, left panel), cytotoxic CD8^+^ T cells (D, middle panel) and immune infiltration scores (D, right panel). E, Correlations of STAT3 gene expression with the proportion of effector memory CD8^+^ T cells (E, left panel), cytotoxic CD8^+^ T cells (E, middle panel) and immune infiltration scores (E, right panel). F, Correlations of TET2/3 and STAT1/3 gene expression with the proportion of M2 macrophage. G, Correlations of STAT1/3 gene expression with the expression of CD274.


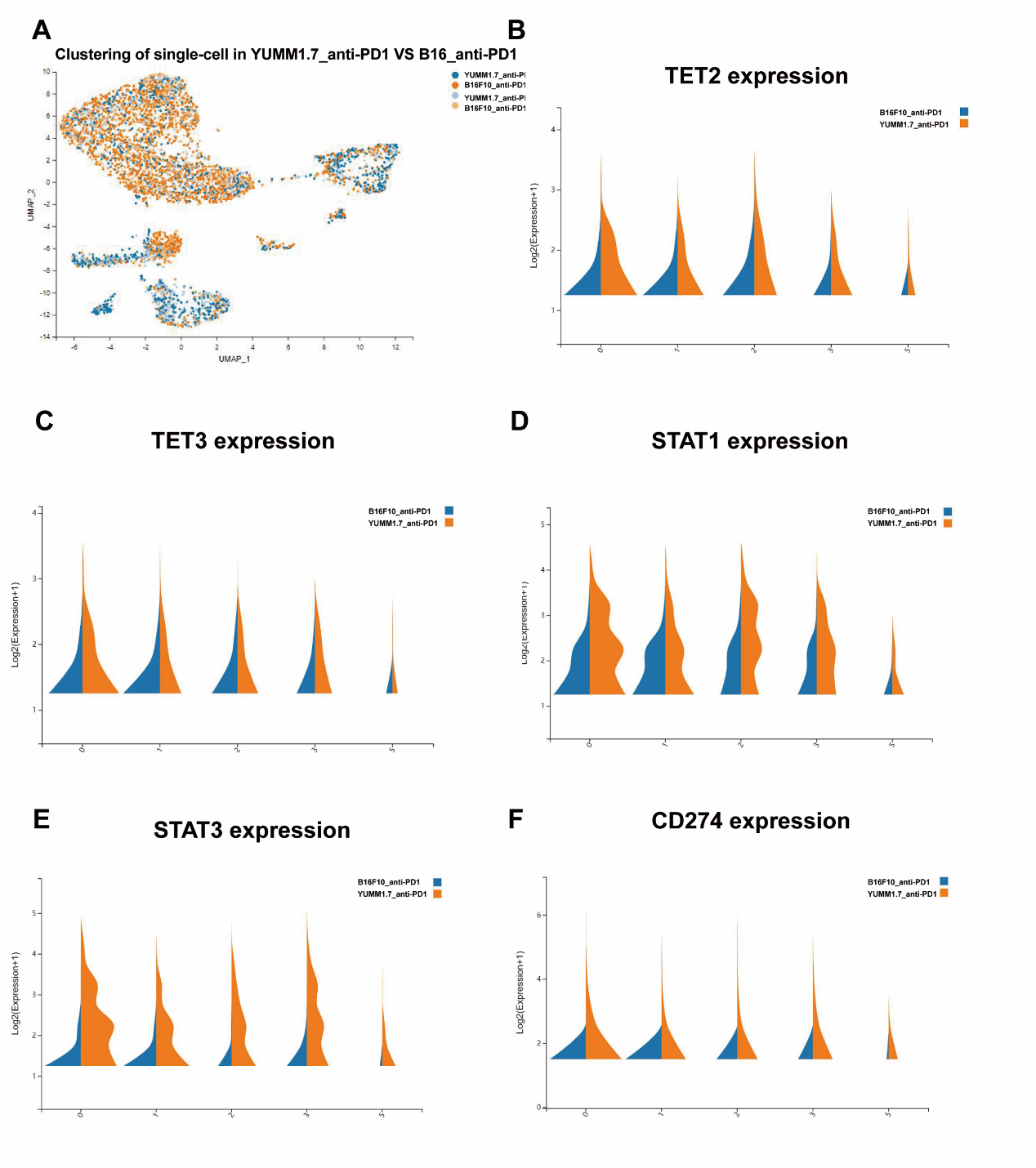


**Supplementary Figure 6. TET2/3-STAT1/3-CD274 signaling was upregulated in tumors of mice that responded to anti-PD1 treatment.** A, Sample source of single-cell cluster constituents. B-F, Gene expression in melanoma clusters after the indicated treatments. Multiple experimental data are counted and presented according to the statistical methods, and an asterisk (*) indicates the degree of significant difference.


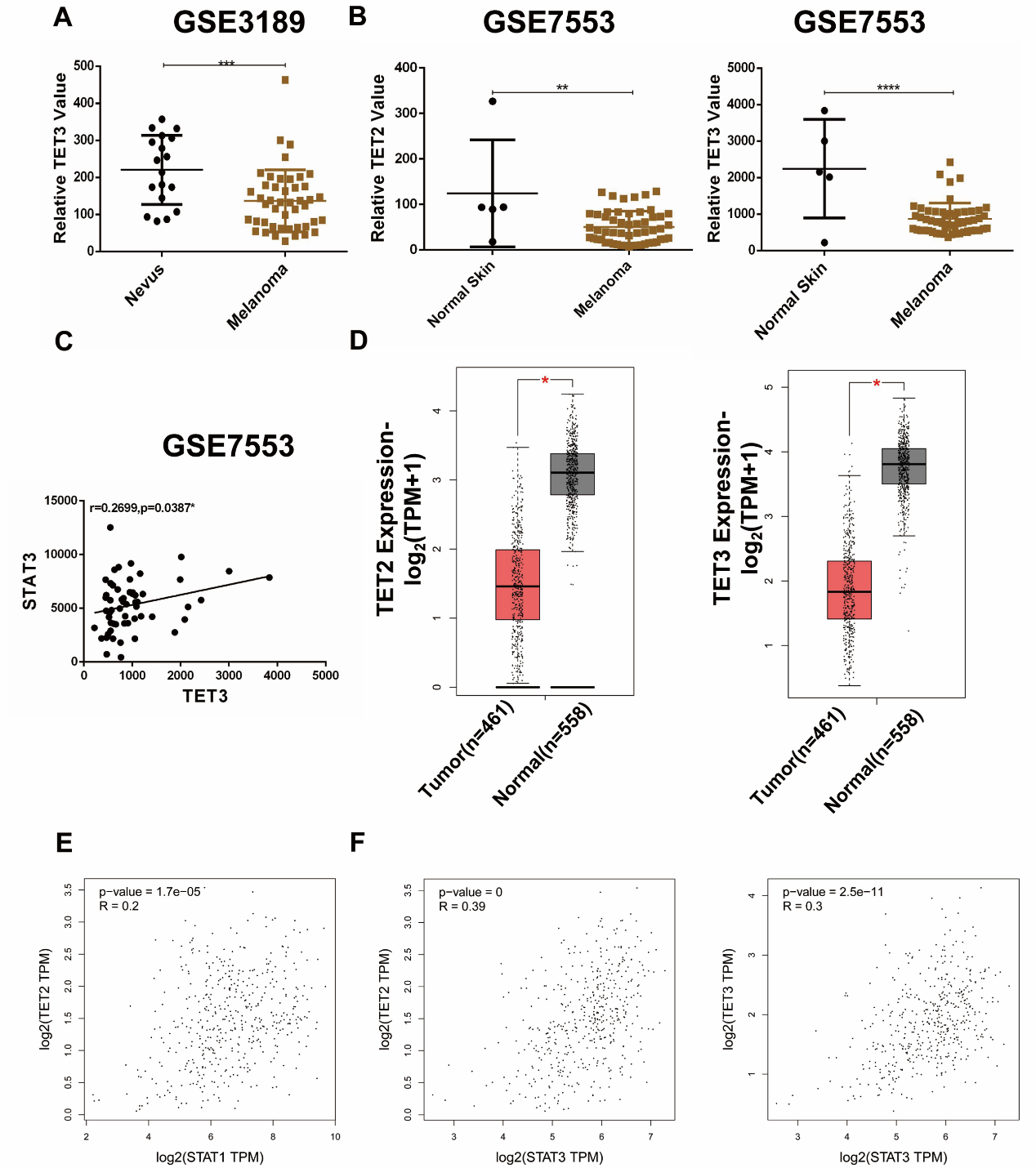


**Supplementary Figure 7.** T**he status of TET2/3-STAT-CD274 expression in melanoma patients.** A-B, Data from the GEO database (GSE3189 and GSE7553) were applied to analyze the expression of TET in melanoma patients. C, Data from the GEO database (GSE7553) were applied to analyze the correlation of STAT3 gene expression with the expression of TET3. D, Data from the TCGA/GTEx database were applied to analyze the expression of TET in melanoma patients. E-F, Data from the TCGA/GTEx database were applied to analyze the correlation of STAT1/3 gene expression with the expression of TET2/3. Multiple experimental data are counted and presented according to the statistical methods, and an asterisk (*) indicates the degree of significant difference.


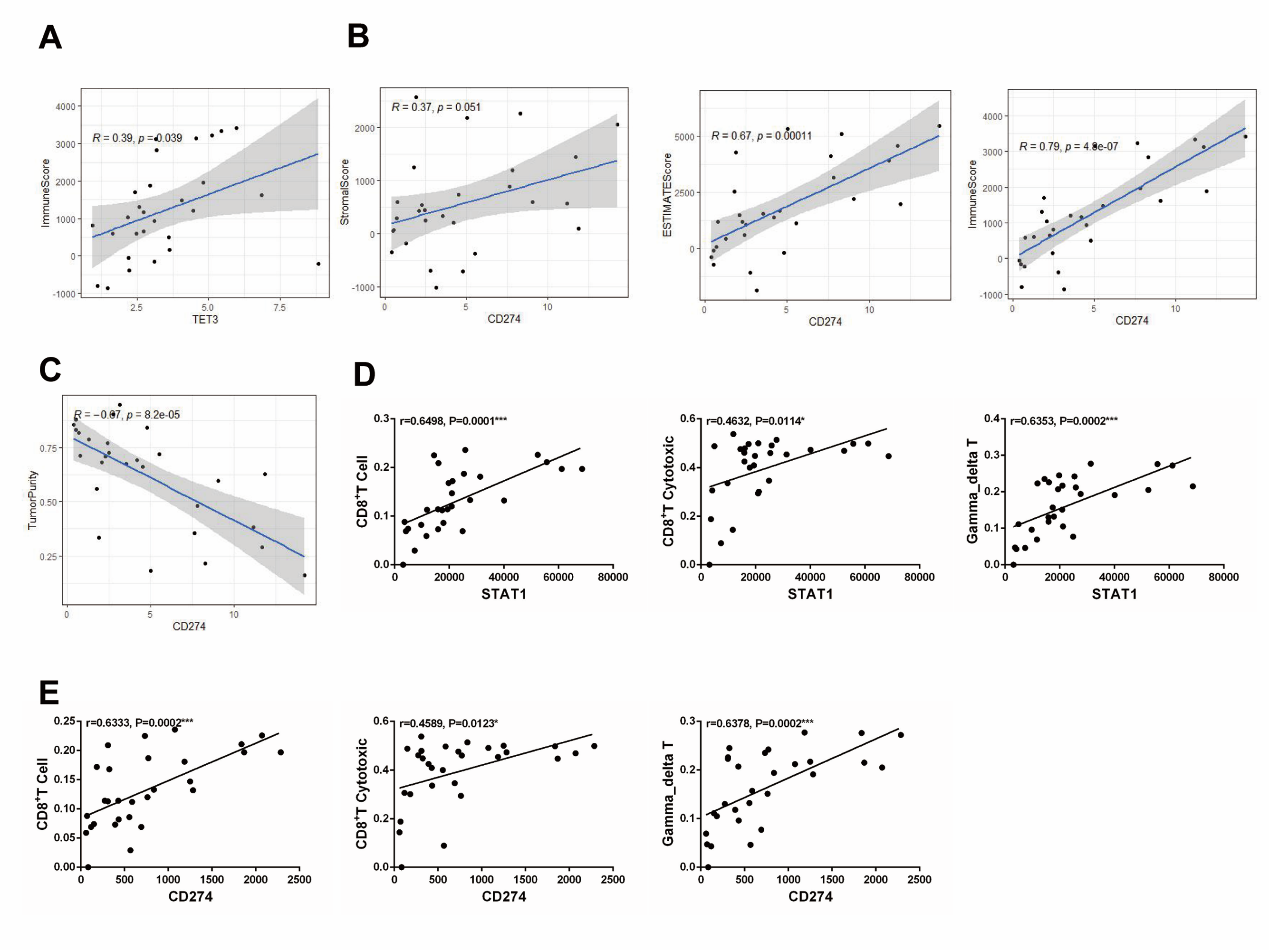


**Supplementary Figure 8. The correlation of STAT-CD274 signaling with immune microenvironment.** A, Data from the GEO database (GSE 91061) were analyzed by the ESTIMATE algorithm to characterize the correlations between TET3 expression and immune scores. B-C, Data from the GEO database (GSE 91061) were analyzed by the ESTIMATE algorithm to characterize the correlations between CD274 expression and stromal scores (B, left panel), estimate scores (B, middle panel), immune scores (B, right panel) or tumor purity (C). D, Data from the GEO database (GSE 91061) were analyzed by the ImmuneCellAI-human method to characterize the immune microenvironment of melanoma tissue. Correlations between STAT1 expression and relative proportions of CD8^+^T cells (D, left panel), cytotoxic CD8^+^ T cells (D, middle panel) and γδ T cells (D, right panel) in the tumor microenvironment. E, Data from the GEO database (GSE 91061) were analyzed by the ImmuneCellAI-human method to characterize the immune microenvironment of melanoma tissue. Correlations between CD274 expression and relative proportions of CD8^+^ T cells (E, left panel), cytotoxic CD8^+^ T cells (E, middle panel) and γδ T cells (E, right panel) in the tumor microenvironment. Data are expressed as the mean (PD n=19, PRCR n=9) ± S.D. Multiple experimental data are counted and presented according to the statistical methods, and an asterisk (*) indicates the degree of significant difference.


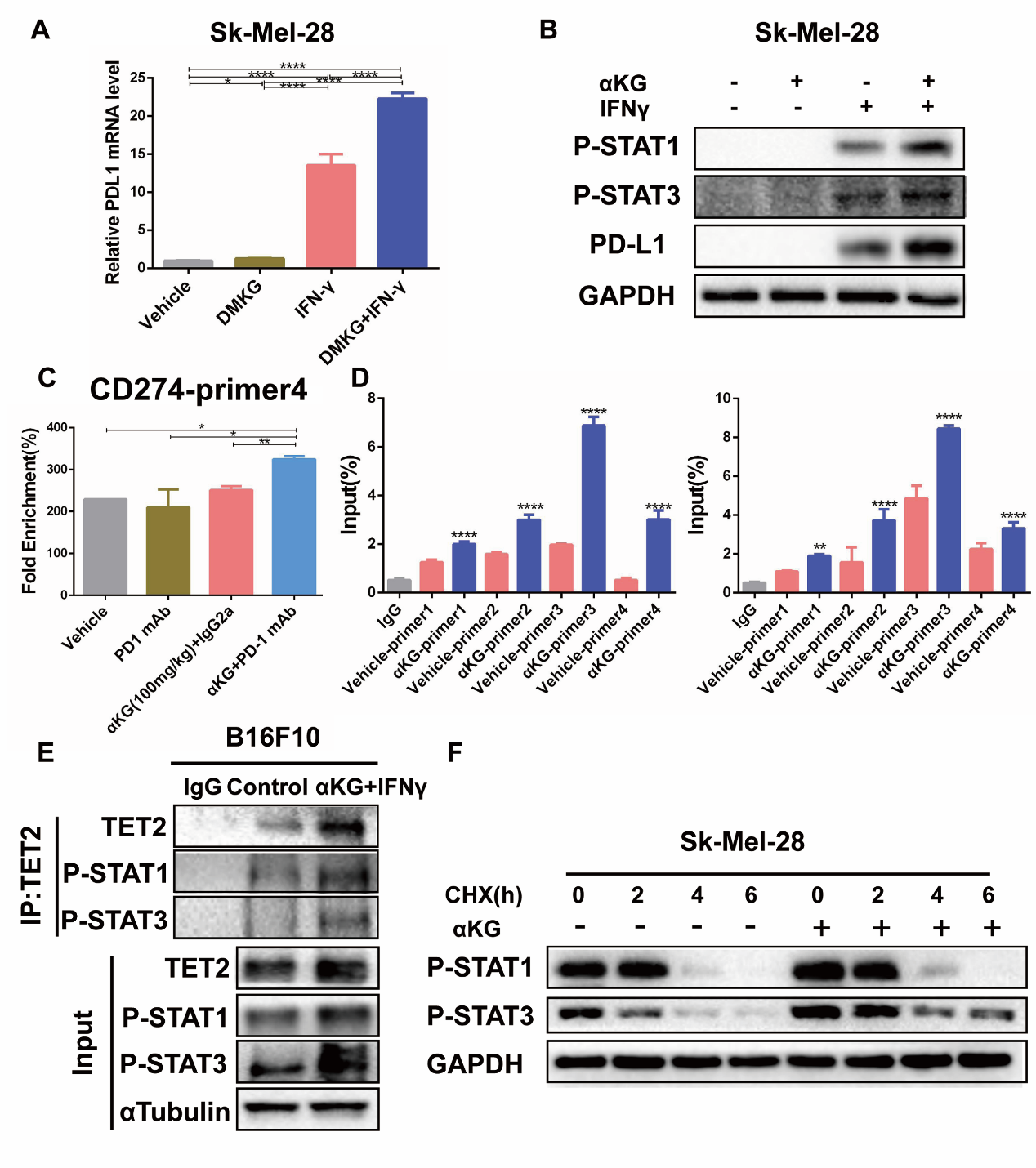


**Supplementary Figure 9. α-ketoglutarate upregulated the expression of IFNγ-induced STAT1/3-PDL1 in melanoma cells.** A-B, extraction of mRNA and protein from treated melanoma cells, and qRT–PCR (A) and immunoblotting (B) were then performed. C, 5-hmC levels of PD-L1 promoters in B16F10 tumor-bearing mouse tissues after the designated treatments. D, ChIP-qPCR was performed to detect the binding of STAT1 (D, left panel) or STAT3 (D, right panel) to PD-L1 promoter sites in B16F10 cells with or without α-KG treatment. E, Immunoprecipitation analysis of TET2 with p-STAT1/3 in B16F10 cells receiving the designated treatments. F, P-STAT1/3 protein levels at different time points after IFNγ stimulation in Sk-Mel-28 cells treated with CHX (100 mg/mL) or α-KG (200 μm). Multiple experimental data (n=3) are counted and presented according to the statistical methods, and an asterisk (*) indicates the degree of significant difference.

**Supplementary Tables:**

**Supplementary Table 1. The primers used in the PCR reaction and annealing**

| Gene name | Sequence (5'to3') | Direction |
| --- | --- | --- |
| Pd-l1 (Human) | GCTGCACTAATTGTCTATTGGG | Forward |
| Pd-l1 (Human) | CACAGTAATTCGCTTGTAGTCG | Reverse |
| Pd-l1 (Mouse) | TGAGCAAGTGATTCAGTTTGTG | Forward |
| Pd-l1 (Mouse) | CATTTCCCTTCAAAAGCTGGTC | Reverse |
| PD-L1 (-64~-273 bp primer 5) | CTATAGACCAGAGACTCACCT | Forward |
| PD-L1 (-64~-273 bp primer 5) | GAAGCCTTGTCCAACCTC | Reverse |
| PD-L1 (-296~-535 bp primer 4) | GTGGGAGCTGTAGAGGAAG | Forward |
| PD-L1 (-296~-535 bp primer 4) | TGCTACTGAGAGGCTGTC | Reverse |
| PD-L1 (-683~-871 bp primer 3) | GTCATGTCAAGACTGTCAC | Forward |
| PD-L1 (-683~-871 bp primer 3) | GTAATGAGGACCCGAATTTC | Reverse |
| PD-L1 (-934~-1200 bp primer 2) | CTGTGGAGTTCCCAAGGT | Forward |
| PD-L1 (-934~-1200 bp primer 2) | CTAGAAAGTAGGTGTGTCTGTA | Reverse |
| PD-L1 (-1223~-1500 bp primer 1) | ACATTTGGAAGAGGGACTATC | Forward |
| PD-L1 (-1223~-1500 bp primer 1) | TCACTGTTGGACTTCAAGG | Reverse |
| Actb (Mouse) | GGGAAATCGTGCGTGAC | Forward |
| Actb (Mouse) | AGGCTGGAAAAGAGCCT | Reverse |
| Actb (Human) | CTACCTCATGAAGATCCTCACCGA | Forward |
| Actb (Human) | TTCTCCTTAATGTCACGCACGATT | Reverse |

**Supplementary Table 2. Clinical characteristics of melanoma patients treated with anti-PD-1 mAb after surgery, related to Supplementary Figure 2E.**

| **Patient No.** | **Classification** | **Gender** | **Age** | **Tumor types** | **TNM stage** | **Response** | **Primary lesion** |
| --- | --- | --- | --- | --- | --- | --- | --- |
| 1 | Non-responders | male | 71 | Melanoma | T3bN3M1a | PD | Left Plantar |
| 2 | Responders | female | 70 | Melanoma | T4bN2bM0 | SD | Right foot |
| 3 | Non-responders | female | 52 | Melanoma | T4bN3Mx | PD | Right Plantar |
| 4 | Non-responders | female | 66 | Melanoma | T2bN1M0 | PD | Right Plantar |
| 5 | Non-responders | female | 72 | Melanoma | T3bN3cM0 | PD | Right calf |
| 6 | Responders | female | 58 | Melanoma | TxN3M1b | SD | Left foot |
| 7 | Non-responders | female | 65 | Melanoma | T3aNxM0 | PD | Right Plantar |
| 8 | Non-responders | female | 64 | Melanoma | T4aNxMx | PD | Left Plantar |
| 9 | Responders | female | 71 | Melanoma | T3bN3Mx | SD | Right heel |
| 10 | Responders | female | 58 | Melanoma | T4bN1aM0 | SD | Right Plantar |
| 11 | Responders | female | 64 | Melanoma | T4bN0M0 | SD | Right heel |
| 12 | Non-responders | female | 56 | Melanoma | T2bN1M0 | PD | Right heel |
| 13 | Non-responders | male | 62 | Melanoma | T4bN0M0 | PD | Right heel |
| 14 | Responders | male | 45 | Melanoma | T4bN1aM0 | SD | Left Plantar |
| 15 | Responders | female | 50 | Melanoma | T3bN3M1a | SD | Left Plantar |
| 16 | Responders | female | 74 | Melanoma | T4bN1aM0 | SD | Left toe |
| 17 | Responders | female | 68 | Melanoma | T3bN2bM1c | SD | Right heel |
| 18 | Non-responders | male | 47 | Melanoma | T3bN3M0 | PD | Back |

Patients were stratified into response groups based on RECIST 1.1 criteria. Patients with non-recurrence > 3 months were classified as responders and SD (stable disease), while patients with SD ≤ 3 months were classified as non-responders and PD (progressive disease). SD, stable disease; PD, progressive disease. TNM stage based on the 8th Edition AJCC Cancer Stage Classification.
