# Supplementary material for: Supplementation with α-ketoglutarate improved the efficacy of anti-PD1 melanoma treatment through epigenetic modulation of PD-L1
Source: Cell Death Dis. 2023 Feb 28;14(2):170. doi: 10.1038/s41419-023-05692-5 (PMC9974984; doi:10.1038/s41419-023-05692-5)

**Original membranes**

The original western blot results in Fiure5A

TET2


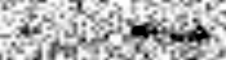


TET3


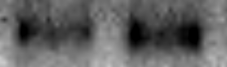


PD-L1


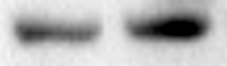


α-Tubulin


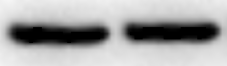


The original western blot results in Fiure5B

TET2


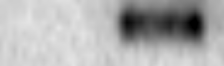


TET3


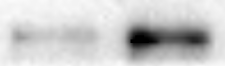


PD-L1


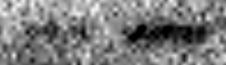


α-Tubulin


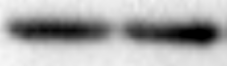


The original western blot results in Fiure5D

P-STAT1


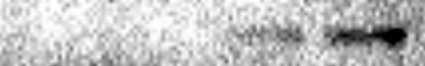


P-STAT3


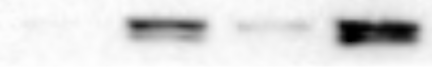


PD-L1


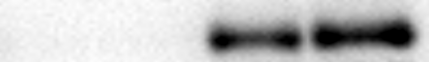


α-Tubulin


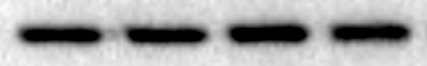


The original western blot results in Fiure5F

TET2


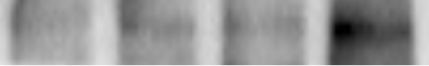


TET3


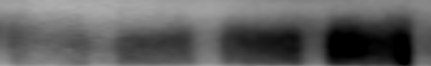


P-STAT1


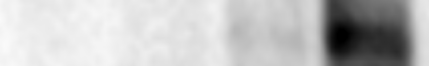


P-STAT3


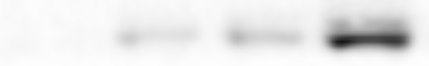


PD-L1


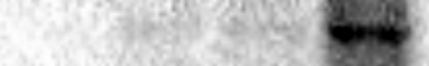


GAPDH


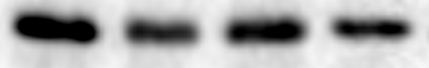


The original western blot results in Fiure6E

TET3


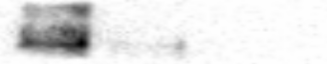


P-STAT1


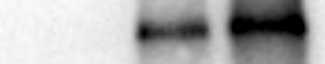


P-STAT3


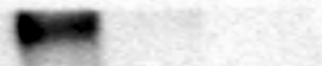


TET3


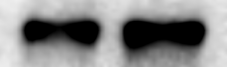


P-STAT1


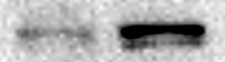


P-STAT3


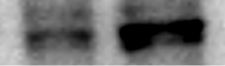


GAPDH


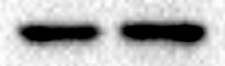


The original western blot results in Fiure6F

P-STAT1


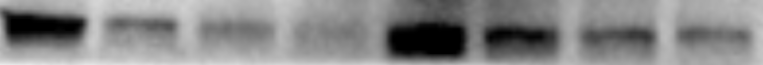


P-STAT3


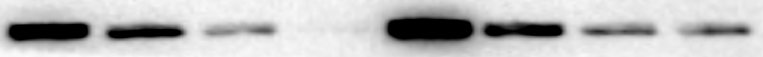


GAPDH


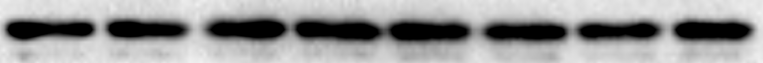


The original western blot results in Fiure6G

TET2(left panel)


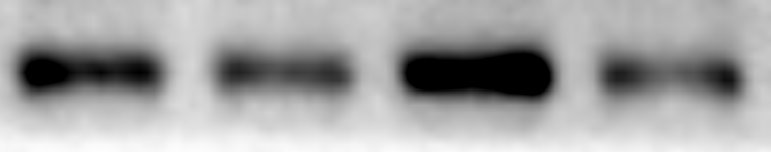


P-STAT1(left panel)


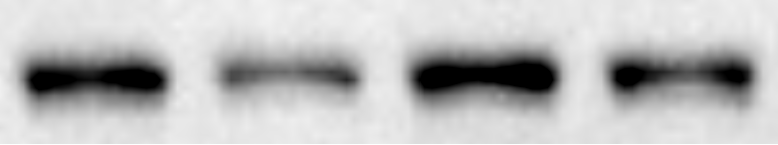


P-STAT3(left panel)


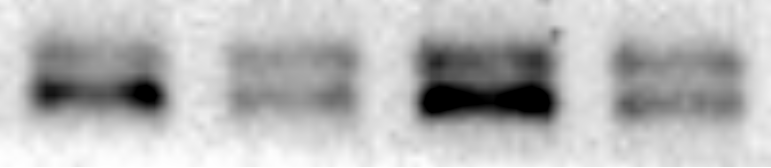


PD-L1(left panel)


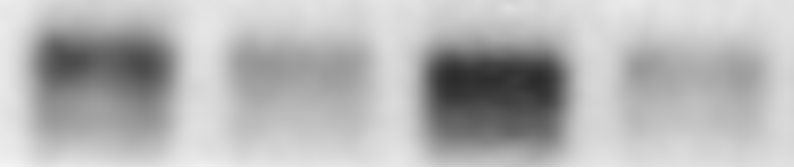


GAPDH(left panel)


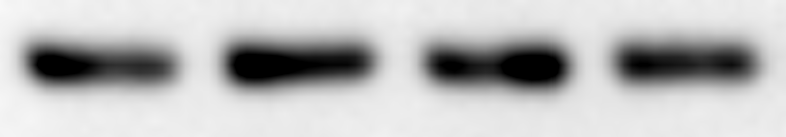


TET3(right panel)


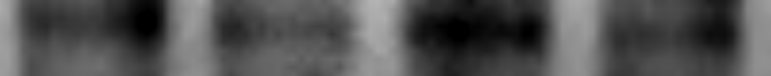


P-STAT1(right panel)


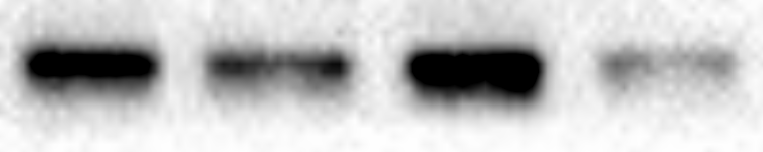


P-STAT3(right panel)


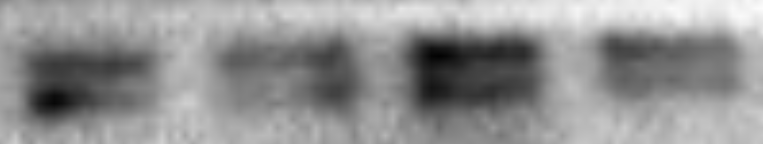


PD-L1(right panel)


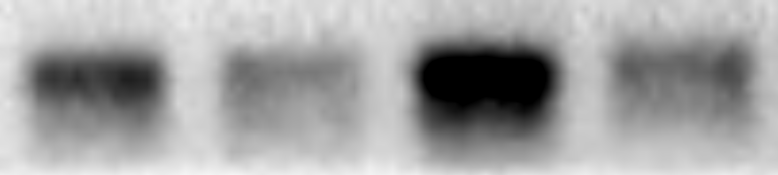


GAPDH(right panel)


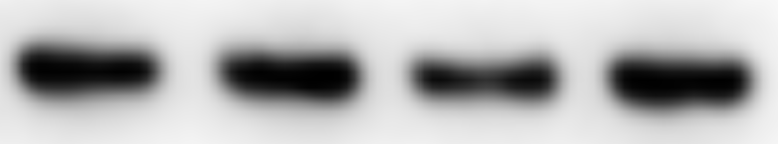


The original western blot results in sFiure5A

H3K9me3


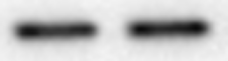


H3K27me3


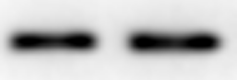


Histone H3


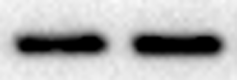


The original western blot results in sFiure5B

H3K9me3


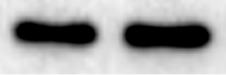


H3K27me3


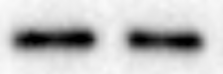


Histone H3


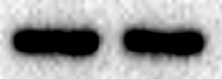


The original western blot results in sFiure9B

P-STAT1


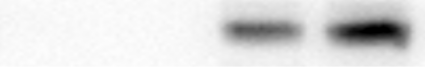


P-STAT3


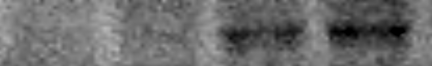


PD-L1


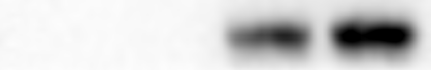


GAPDH


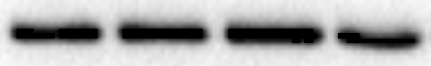


The original western blot results in sFiure9E

TET2


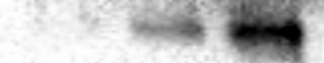


P-STAT1


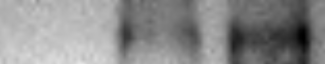


P-STAT3


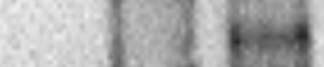


TET2


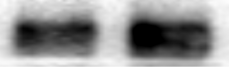


P-STAT1


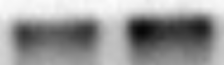


P-STAT3


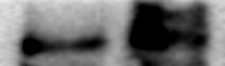


αTubulin


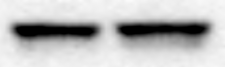


The original western blot results in sFiure9F

P-STAT1


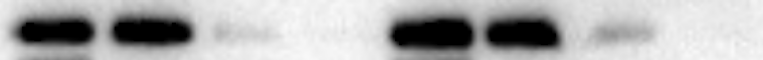


P-STAT3


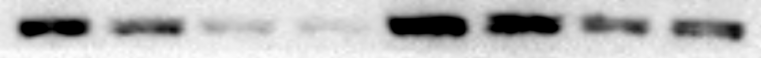


GAPDH


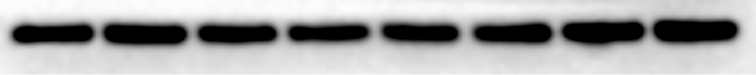

Supplement: Supplementary file 2 — Original western blots [file 41419_2023_5692_MOESM2_ESM.docx]
